# β-Hexosaminidases Along the Secretory Pathway of *Nicotiana benthamiana* Have Distinct Specificities Toward Engineered Helminth N-Glycans on Recombinant Glycoproteins

**DOI:** 10.3389/fpls.2021.638454

**Published:** 2021-03-17

**Authors:** Nicolò Alvisi, Kim van Noort, Sarlita Dwiani, Nathan Geschiere, Octavina Sukarta, Koen Varossieau, Dieu-Linh Nguyen, Richard Strasser, Cornelis H. Hokke, Arjen Schots, Ruud H. P. Wilbers

**Affiliations:** ^1^Laboratory of Nematology, Plant Sciences Group, Wageningen University and Research, Wageningen, Netherlands; ^2^Department of Parasitology, Leiden University Medical Center, Leiden, Netherlands; ^3^Department of Applied Genetics and Cell Biology, University of Natural Resources and Life Sciences, Vienna, Austria

**Keywords:** *Nicotiana benthamiana*, β-hexosaminidases, N-glycosylation, glyco-engineering, LacdiNAc

## Abstract

Secretions of parasitic worms (helminths) contain a wide collection of immunomodulatory glycoproteins with the potential to treat inflammatory disorders, like autoimmune diseases. Yet, the identification of single molecules that can be developed into novel biopharmaceuticals is hampered by the limited availability of native parasite-derived proteins. Recently, pioneering work has shown that helminth glycoproteins can be produced transiently in *Nicotiana benthamiana* plants while simultaneously mimicking their native helminth N-glycan composition by co-expression of desired glycosyltransferases. However, efficient “helminthization” of N-glycans in plants by glyco-engineering seems to be hampered by the undesired truncation of complex N-glycans by β-*N*-acetyl-hexosaminidases, in particular when aiming for the synthesis of N-glycans with antennary GalNAcβ1-4GlcNAc (LacdiNAc or LDN). In this study, we cloned novel β-hexosaminidase open reading frames from *N. benthamiana* and characterized the biochemical activity of these enzymes. We identified HEXO2 and HEXO3 as enzymes responsible for the cleavage of antennary GalNAc residues of N-glycans on the model helminth glycoprotein kappa-5. Furthermore, we reveal that each member of the HEXO family has a distinct specificity for N-glycan substrates, where HEXO2 has strict β-galactosaminidase activity, whereas HEXO3 cleaves both GlcNAc and GalNAc. The identification of HEXO2 and HEXO3 as major targets for LDN cleavage will enable a targeted genome editing approach to reduce undesired processing of these N-glycans. Effective knockout of these enzymes could allow the production of therapeutically relevant glycoproteins with tailor-made helminth N-glycans in plants.

## Introduction

Over the last 50 years, inflammatory conditions such as autoimmune diseases have dramatically risen in industrialized countries. For example, the prevalence of autoimmune type 1 diabetes (Patterson et al., 2009), inflammatory bowel disease (Economou and Pappas, 2008), and asthma (Asher et al., 2006) has increased in Europe. Autoimmune diseases are exaggerated immune responses against harmless self-antigens, and the worldwide occurrence of autoimmune disease is correlated with several “western lifestyle” behaviors (Thorburn et al., 2014). For example, improved hygienic conditions are thought to impact the development of our immune system. In particular, the “old friends” hypothesis links this altered immune development with the absence of parasitic worm (helminth) infections (Stiemsma et al., 2015). It is believed that co-evolution of humans and helminths has resulted in a mutually beneficial balance.

Helminths are a heterogeneous group of parasitic worms that chronically infect humans and other animals. Helminths stably live inside the host due to their ability to modulate immune responses of the host. Helminths elicit a modified type 2 immune response (Th2), enhance the generation of regulatory T cells (Tregs), and stimulate the production of anti-inflammatory cytokines (Finlay et al., 2014; Zaph et al., 2014). The induction of these immunoregulatory mechanisms is thought to be responsible for a protective effect against many inflammatory conditions (Finlay et al., 2014; Helmby, 2015). For example, several studies in animal models showed the protective effect of *Schistosoma mansoni* infection against colitis (Elliott et al., 2003), airway inflammation (Mangan et al., 2006), encephalomyelitis (Sewell et al., 2003), and diabetes (Cooke et al., 1999; Hussaarts et al., 2015). Due to these findings, many helminths are under investigation for their potential to treat autoimmune diseases, allergies, and metabolic disorders.

Upon infection, helminths excrete/secrete a variety of glycoproteins and gycolipids (ES products) that play a vital role in immunomodulation (reviewed in Maizels et al., 2018). For this reason, ES products are considered valuable candidates for therapy of inflammatory conditions. Unfortunately, the extraction and purification of single ES products is not sustainable for functional characterization and subsequent therapeutic application, since it is practically impossible to obtain large quantities of worm material. The detailed characterization of ES products therefore relies on the production of glycoproteins in a recombinant expression system. However, the glycan composition of helminth glycoproteins is highly diverse (reviewed in Hokke and van Diepen, 2017), and studies with ES products revealed that N-glycosylation can be crucial for their biological activity. For example, the N-glycans of *S. mansoni* egg-secreted omega-1, which carry Lewis X, are necessary for its interaction with immune cells and immunomodulatory properties (Everts et al., 2012; Wilbers et al., 2017), which can be harnessed to improve metabolic homeostasis in obese mice (Zande et al., 2021). For this reason, an expression system is preferred that not only produces the desired glycoprotein in large quantities, but at the same time allows the engineering of the native N-glycan composition.

Plants are an attractive expression system for the production of such “helminthized” glycoproteins. Plants offer advantages over other expression systems in terms of scalability, production speed and costs, and product quality (Stoger et al., 2014; Nandi et al., 2016; Mir-Artigues et al., 2019). When it comes to engineering of helminth N-glycans, *Nicotiana benthamiana* plants also offer unprecedented flexibility. First of all, their limited endogenous glycome shares several characteristics of helminth N-glycans, such as the lack of sialylation and the presence of β1,2-xylose and/or core α1,3-fucose. Secondly, plants tolerate changes in their glycosylation pathway, allowing the modification of recombinant glycoproteins in a controlled and uniform manner (Bosch et al., 2013). For example, typical mammalian glycan modifications that are shared with certain helminth N-glycans can be synthesized in *N. benthamiana*, such as core α1,6-fucosylation (Castilho et al., 2011a; Wilbers et al., 2016), additional branching (Castilho et al., 2011b), extension of antennae with β1,4-galactose, or Lewis X (Bakker et al., 2001; Rouwendal et al., 2009; Castilho et al., 2011a; Wilbers et al., 2017). In addition to humanized N-glycan engineering, several complex helminth glycan motifs, like LDN, LDN-F, and F-LDN-F, have previously been synthesized in *N. benthamiana* (Wilbers et al., 2017; van Noort et al., 2020). Finally, the availability of transgenic *N. benthamiana* plants that lack the typical plant sugar residues β1,2-xylose and/or core α1,3-fucose enable even greater flexibility of glyco-engineering strategies (Strasser et al., 2008; Jansing et al., 2019). Altogether, this makes *N. benthamiana* a highly versatile expression system for the production of helminth glycoproteins with their native N-glycan composition.

Recently, we have exploited *N. benthamiana* to produce *S. mansoni* egg-secreted omega-1 and kappa-5 and mimicked their native N-glycan composition [Lewis X and LDN(-F), respectively] (Wilbers et al., 2017). However, “helminthization” of N-glycans in plants by glyco-engineering still faces some bottlenecks, in particular the synthesis of N-glycans with antennary GalNAcβ1-4GlcNAc (LDN) motifs, which are present on the N-glycans of native kappa-5 (Meevissen et al., 2011), but not omega-1. LDN-glycans were synthesized in *N. benthamiana* by transiently co-expressing kappa-5 and a β1,4-*N-*acetyl-galactosaminyltransferase from *Caenorhabditis elegans* (CeGalNAcT), but only a relatively small fraction of N-glycans carried terminal LDN on a single antenna. It was hypothesized that plant β-hexosaminidases (HEXOs) were responsible for the cleavage of the LDN motif (Wilbers et al., 2017).

**FIGURE 1:**
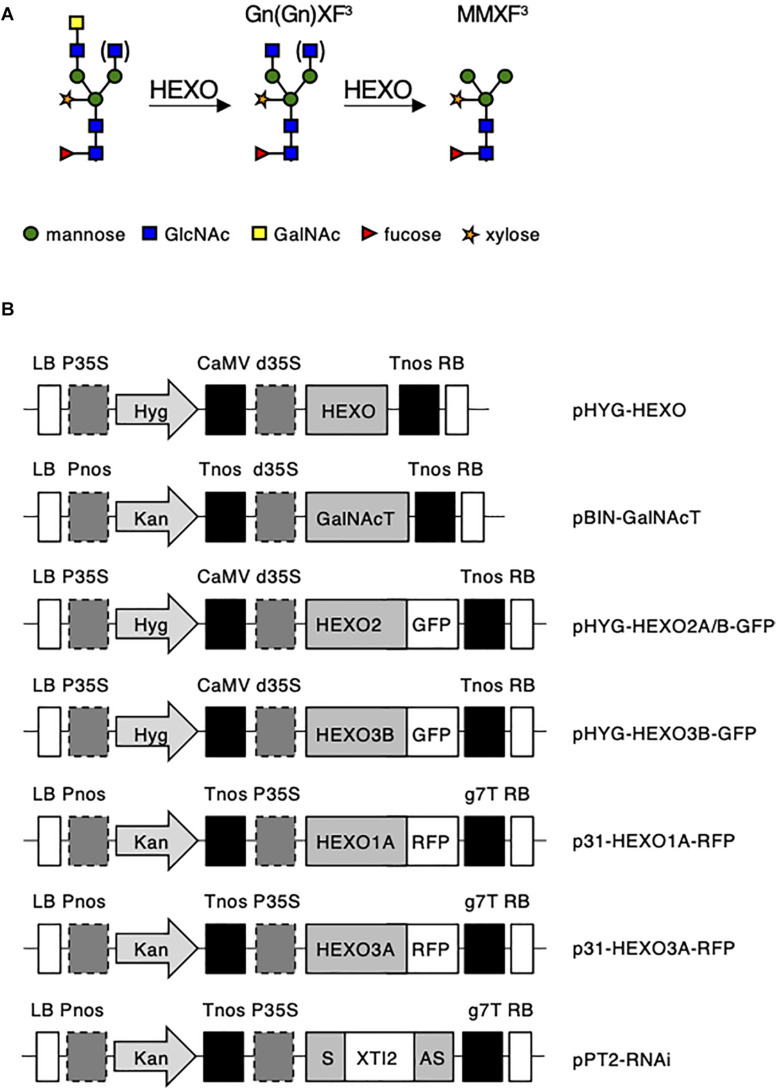
**(A)** Schematic illustration of proposed plant HEXO activity on complex LDN-carrying N-glycans. **(B)** Schematic overview of the expression vectors used in this study. LB, left border; Pnos, nopaline synthase gene promoter; Hyg, hygromycin B phosphotransferase gene; Kan, neomycin phosphotransferase 2 gene; Tnos, nopaline synthase gene terminator; CaMV, cauliflower mosaic virus; d35S, dual cauliflower mosaic virus 35S gene promoter; P35S, cauliflower mosaic virus 35S gene promoter; HEXO, *N. benthamiana* HEXO ORF; GalNAcT, β1,4-*N*-acetyl-galactosaminyltransferase from *Caenorhabditis elegans;* GFP, green fluorescent protein; RFP, red fluorescent protein; XYLT, Arabidopsis thaliana b1,2-xylosyltransferase ORF; S: HEXO RNAi sequence in sense orientation; XTI2, intron 2 from *A. thaliana* XYLT; AS, HEXO RNAi sequence in antisense orientation; g7T, Agrobacterium gene 7 terminator; and RB, right border.

Plant HEXOs are a class of enzymes capable of removing terminal GlcNAc residues from complex N-glycans (Altmann et al., 1995) and are expected to be able to cleave GalNAc as well. Three different members comprise the HEXO family (HEXO1, HEXO2, and HEXO3) in *Arabidopsis thaliana* and *N. benthamiana* (Gutternigg et al., 2007; Strasser et al., 2007; Liebminger et al., 2011; Shin et al., 2017). Enzymatic activity for the HEXOs from Arabidopsis has been analyzed with chemically synthesized substrates (Gutternigg et al., 2007; Strasser et al., 2007), which showed a strong preference of all three HEXOs for GlcNAc substrates over GalNAc substrates (Strasser et al., 2007). Both studies also showed that HEXO1 and HEXO3 are acting on the complex plant N-glycan structure GnGnXF^3^ (Figure 1A), whereas HEXO2 displayed high activity toward chitooligosaccharides [chitotriose or (GlcNAc)_3_]. Evaluation of HEXOs in the plant (*A. thaliana* and *N. benthamiana*) revealed that HEXO1 is secreted into the vacuole where it produces paucimannosidic N-glycans on vacuolar glycoproteins (Strasser et al., 2007; Liebminger et al., 2011; Castilho et al., 2014). Arabidopsis HEXO2 is found at the plasma membrane and does not appear to play a role in the processing of endogenous N-glycans (Strasser et al., 2007; Liebminger et al., 2011). HEXO3 is localized in the apoplast and is the major contributor to the formation of paucimannosidic N-glycans on secreted glycoproteins in *A. thaliana* and *N. benthamiana* (Strasser et al., 2007; Liebminger et al., 2011; Castilho et al., 2014). So far, not all HEXO orthologs from allotetraploid *N. benthamiana* have been identified, and no evidence has been reported that HEXOs are able to cleave GalNAc residues from N-glycans *in vivo*.

In this study we investigated the role of *N. benthamiana* HEXOs on the cleavage of engineered LDN-carrying N-glycans. We used kappa-5 as model protein since the native parasite protein carries LDN-glycans. We demonstrated that HEXO2 and HEXO3, but not HEXO1, are responsible for cleaving the LDN motif of N-glycans on apoplast glycoproteins. Furthermore, we show that HEXO2 can act on N-glycans of apoplast glycoproteins as a membrane-bound enzyme, while HEXO3 is secreted into the apoplast. Interestingly, HEXO2 was only able to cleave terminal GalNAc residues, whereas HEXO3 truncated N-glycans completely to paucimannosidic structures. Unfortunately, attempts to improve LDN synthesis by transient knock-down by RNAi-mediated silencing were unsuccessful. In conclusion, this study reveals distinct N-glycan substrate specificities for the different HEXO family members of *N. benthamiana* and the ability of HEXO2 to process non-endogenous N-glycans. The identification of HEXO2 and HEXO3 as major targets for LDN cleavage enables targeted knockout of these enzymes by genome editing approaches and could further optimize *N. benthamiana* as production platform for “helminthized” glycoproteins.

## Materials and Methods

### Identification of HEXO Orthologs in *N. benthamiana*

The *A. thaliana* HEXO1 (At3g55260), HEXO2 (At1g05590), HEXO3 (At1g65590) amino acid sequences were used to identify corresponding genes in the Sol Genomics Network draft genome for *N. benthamiana* (v1.0.1; Bombarely et al., 2012). Primers were designed to amplify the full-length open reading frames (ORF) from *N. benthamiana* cDNA (Supplementary Table 1). Thereto, mRNA was isolated from 5-week-old *N. benthamiana* plants using the SV Total RNA Isolation System (Promega, Leiden, Netherlands), and cDNA was synthesized using oligo(dT) primers with GoScript reverse transcriptase (Promega, Leiden, Netherlands). Additionally, 3′ RACE PCR from the poly-A tail was performed for HEXO2 orthologs. We used an oligo(dT) primer that was modified with a 3′ adaptor sequence for cDNA synthesis as described above. In a second round of PCR we used a gene specific HEXO2 primer and adaptor primer to amplify the 3′ end of HEXO2 orthologs (Supplementary Table 1). Full-length HEXO ORFs were then PCR amplified, cloned into pCR2.1-TOPO (Fisher Scientific, Landsmeer, Netherlands), and sequenced at Macrogen (Amsterdam, Netherlands).

### Construction of Expression Vectors

All HEXO ORFs were reamplified and subcloned into the plant expression vector pHYG (Westerhof et al., 2012) via 5′ *Nco*I/*Bsp*HI and 3′ *Kpn*I/*Bsr*GI restriction sites (Westerhof et al., 2012). Additionally, fusions of HEXO2A, HEXO2B, and HEXO3B with green fluorescent protein (GFP) were constructed in the pHYG plant expression vector. The HEXO ORFs were reamplified by PCR to introduce a GGGGS-linker and a *Nhe*I restriction site at the 3′ end in order to clone the HEXOs in frame with a C-terminal GFP fragment in pHYG. Fusion proteins of HEXO1A and HEXO3A with red fluorescent protein (RFP) in the p31 vector were included in this study (Shin et al., 2017), and mCherry-tagged formin was used as a marker for membrane localization (Favery et al., 2004).

To determine HEXO activity toward endogenous plant N-glycans we co-expressed HEXO genes with the carrier glycoprotein kappa-5 (in pHYG) and silencing suppressor p19 (in pBIN61) as described before (Wilbers et al., 2017). For screening the ability of HEXOs to cleave GalNAc residues we used β1,4-*N*-acetyl-galactosaminyltransferase from *C. elegans* (CeGalNAcT) in the pBINPLUS expression vector to synthesize β1,4-galactosamine (GalNAc) extended branches (Wilbers et al., 2017). In order to transiently silence HEXO genes, a sense–intron–antisense hairpin construct targeting amino acids 136–208 of HEXO3A (pPT2-HEXO3-RNAi) was used (Shin et al., 2017). A sense-intron-antisense construct targeting both HEXO2 and HEXO3 was synthetically constructed at GeneArt and subcloned into the pPT2-HEXO3-RNAi via *Xba*I/*Bam*HI sites (pPT2-HEXO2/3-RNAi). A HEXO2 subfragment (targeting amino acids 71–144 of HEXO2B) was subcloned into pPT2-HEXO3-RNAi via *Spe*I/*Bgl*II sites (pPT2-HEXO2-RNAi). These hairpin constructs target regions that share 94–96% sequence identity between A and B HEXO orthologs (Supplementary Figures 6, 7).

For plant expression, all constructs were transformed into *Agrobacterium tumefaciens* strain MOG101, and a schematic representation of the expression vectors can be found in Figure 1B.

### Agroinfiltration

*Agrobacterium tumefaciens* cultures were grown at 28°C/250 rpm in LB medium (10 g/L peptone140, 10 g/L NaCl, 5 g/L yeast, pH 7.0), with 50 μg/mL kanamycin and 20 μM acetosyringone. After overnight (o/n) incubation, bacterial cultures were centrifuged for 15 min/2880 × *g*. Bacteria were directly resuspended in MMA (1.95 g/L MES, 20 g/L sucrose, 5 g/L MS-salts, 0.2 mM acetosyringone, pH 5.6). *A. tumefaciens* cultures of different constructs were mixed for co-expression, and the final optical density (at 600 nm) of each *A. tumefaciens* culture in the mixture was 0.5. The youngest fully expanded leaves of four to 6-week-old *N. benthamiana* plants were infiltrated at the abaxial side with a needleless syringe. Plants were maintained throughout the experiment in a controlled greenhouse compartment at 20°C and 16 h of light (UNIFARM, Wageningen, Netherlands). Leaves were harvested 3 days post infiltration (dpi) or at 6 dpi when p19 was co-infiltrated.

### Golgi Localization

To determine HEXO subcellular localization, *N. benthamiana* leaves were infiltrated with GFP- or RFP-tagged HEXOs and mCherry-tagged Formin as a plasma membrane marker. To observe fluorescent protein expression and subcellular localization, pictures were acquired with the 63x/1.4 Oil DIC objective on a Zeiss LSM 510 laser scanning microscope (Zeiss, Oberkochen, Germany) using the ZEN-2012 software with scanning speed 9 and average of 16.

### Protein Isolation and Purification

Leaves were harvested, and apoplastic proteins were isolated via apoplast wash. Harvested leaves were submerged in extraction buffer (50 mM phosphate buffer, 0.1 M NaCl, 0,1% v/v Tween-20, pH 8.0), a vacuum (−1,000 mBar) was applied in a plastic desiccator and after 5 min slowly released to infiltrate the leaves with buffer. The apoplast fluid was extracted from the leaves by centrifugation for 10 min/2,000 × *g* (gently rolled into a 10-ml syringe). Remaining intracellular proteins were subsequently isolated from the leaves by homogenization in liquid nitrogen using a metal ball and TissueLyser II (Qiagen, Venlo, Netherlands). Homogenization was then repeated in ice-cold extraction buffer [50 mM phosphate-buffered saline (pH = 8), 100 mM NaCl, 0.1% v/v Tween-20, and 2% w/v immobilized polyvinylpolypyrrolidone (PVPP)] using 2 mL/g fresh weight. Crude extracts were clarified by centrifugation at 16,000 × *g* for 5 min at 4°C. The protein concentration of the apoplast fluid was determined by the Pierce Bicinchoninic Acid Protein Assay (BCA, Fisher Scientific).

Prior to purification of kappa-5, extracted apoplast fluids were passed through Sephadex G25 chromatography columns to exchange extraction buffer for binding buffer (10 mM Sørensen’s phosphate buffer, 0.1 M NaCl, pH 6.0) and subsequently clarified by centrifugation for 5 min/16,000 × *g*. Proteins were then bound to Pierce Strong Cation Exchange Mini Spin Columns (Fisher Scientific, Landsmeer, Netherlands). Kappa-5 was eluted with binding buffer containing 2 M NaCl. Column loading, washing, and elution were done by centrifugation for 5 min/2,000 × *g*. After elution, samples were dialyzed against PBS. The protein concentration of the purified proteins was determined by a BCA assay (Fisher Scientific, Landsmeer, Netherlands).

### SDS-PAGE and Western Blots

Apoplastic proteins and intracellular fractions containing GFP- or RFP-tagged HEXOs were analyzed by western blot. Proteins were run on a NuPAGE 12% Bis-Tris (Fisher Scientific, Landsmeer, Netherlands) under reducing conditions and subsequently transferred to a PVDF membrane by wet blotting in a XCell II^TM^ Blot Module using NuPAGE transfer buffer (Fisher Scientific, Landsmeer, Netherlands). After blotting, the membrane was blocked for 1 h at room temperature (RT) or o/n at 4°C with 5% w/v bovine serum albumin in PBST (PBS containing 0.1% v/v Tween-20). Next, the membrane was incubated for 1 h at RT or o/n at 4°C with horseradish peroxidase (HRP)-conjugated antibodies targeting RFP (Abcam, Cambridge, United Kingdom) or GFP (Miltenyi Biotec, Leiden, Netherlands). The membrane was washed five times with PBST, and the HRP-conjugated antibodies were detected with a 1:1 SuperSignal West Femto:Dura substrate (Fisher Scientific, Landsmeer, Netherlands) in the G:BOX Chemi System (VWR International, Amsterdam, Netherlands).

### Lectin Binding Assays

The presence of terminal GlcNAc or GalNAc residues on the N-glycans of kappa-5 was analyzed with biotinylated *Griffonia simplicifolia* lectin II (GSL-II) or soybean agglutinin (SBA), respectively (Bio-Connect, Huissen, Netherlands). Microtiter plates were coated o/n with apoplast fluids in PBS at a protein concentration of 1 and 10 μg/mL. Plates were blocked with carbohydrate-free blocking buffer (Bio-Connect, Huissen, Netherlands) for 1 h at RT. Plates were then incubated for 1 h at RT with biotinylated lectin at a concentration of 2 μg/mL GSL-II or 5 μg/mL SBA. Subsequently, plates were incubated with avidin-HRP (Fisher Scientific, Landsmeer, Netherlands) for 30 min at RT. After every incubation step, the microtiter plates were washed five times with PBST (PBS containing 0.05% v/v Tween-20). Lectin binding was visualized by adding liquid 3,3′,5,5′-Tetramethylbenzidine (TMB) substrate (Fisher Scientific, Landsmeer, Netherlands), and absorbance was measured at a wavelength of 450 nm while using 655 nm as reference filter in a microplate spectrophotometer (BioRad, Lunteren, Netherlands).

### Glycan Analysis

Glycans of purified kappa-5 were released, purified, and labeled with anthranilic acid as previously described (Wilbers et al., 2017) and analyzed by matrix-assisted laser desorption/ionization time-of-flight mass spectrometry (MALDI-TOF MS). MS spectra were obtained using an Ultraflex II mass spectrometer (Bruker Daltonics) in negative-ion reflection mode. To confirm the presence of terminal GlcNAc or GalNAc residues, N-glycans were treated with β-*N*-acetyl-glucosaminidase from *Streptococcus pneumoniae* (BIOKÉ, Leiden, Netherlands) prior to ZipTip C18 (Millipore BV, Amsterdam, Netherlands) clean-up and subsequent MALDI-TOF MS analysis.

## Results

### Cloning of Novel HEXO Genes From *Nicotiana benthamiana*

To find HEXO orthologs in *N. benthamiana*, we performed a BLAST search against the *N. benthamiana* draft genome (solgenomics v1.0.1) with the amino acid sequences of *A. thaliana* HEXOs (AtHEXO1, AtHEXO2, and AtHEXO3). This BLAST search yielded two genomic scaffolds putatively encoding for HEXO1 (Niben101Scf03794g01004.1 and Niben101Scf10015g07014.1). The first scaffold corresponds to the HEXO1 sequence obtained by Shin et al. (2017), whereas Niben101Scf10015g07014.1 could encode a HEXO1 ortholog that lacks 143 amino acids of the N-terminus compared to the other HEXO1 ortholog and AtHEXO1 (Supplementary Figure 1). In addition, a HEXO1 contig (Niben101Ctg15557g00001.1) is present in the database that could encode these missing 143 amino acids. For HEXO2, three genomic scaffolds were found (Niben101Scf09360g01009, Niben101Scf15928g00003.1, and Niben101Scf01151g00003.1). The first scaffold putatively encodes a full-length HEXO2 ortholog, whereas the other two scaffolds contain the *N*-terminal and *C*-terminal part of a second HEXO2 ortholog, respectively. For HEXO3, two genomic scaffolds were found (Niben101Scf02017g00001 and Niben101Scf02405g02003) of which the first scaffold corresponds to the HEXO3 sequence published by Shin et al. (2017).

To obtain a complete set of orthologous HEXO sequences of *N. benthamiana* (besides the HEXO1A and HEXO3A sequences that were published while this study was ongoing) we PCR-amplified the full-length ORFs of HEXO1, HEXO2, and HEXO3 from leaf cDNA. With this approach, we were able to retrieve two novel HEXO2 ORFs (designated as HEXO2A: Niben101Scf09360g01009 and HEXO2B: combination of Niben101Scf15928-g00003.1 and Niben101Scf01151g00003.1; Supplementary Figure 2). HEXO2A encodes a 548 amino acid protein with 59% sequence identity with AtHEXO2 and has a 46 amino acid truncation compared to AtHEXO2. HEXO2B encodes a 602 amino acid protein with 64% sequence identity to AtHEXO2. Both HEXO2 ORFs seem to lack a signal peptide for secretion (SignalP-5.0 server prediction) and have a 23 amino acid transmembrane region just like AtHEXO2 (TMHMM server prediction), where the catalytic domain faces toward the apoplast.

In addition to these novel HEXO2 ORFs, we successfully amplified HEXO1 and HEXO3 ORFs, of which the majority of sequences corresponded to those previously reported by Shin et al. (2017) (designated as HEXO1A and HEXO3A in this study). Despite several efforts we were not able to obtain a second HEXO1 ortholog. The majority of the retrieved sequences for a second HEXO3 ortholog contained premature stop codons, but we did obtain a single clone that could encode a full-length HEXO3 ortholog (designated as HEXO3B: Niben101Scf02405g02003; Supplementary Figure 3). HEXO3B displays 95% sequence identity with HEXO3A and has a predicted signal peptide for secretion just like HEXO3A. In addition, both HEXO3 orthologs lack a clear transmembrane region (TMHMM server prediction), which is in contrast to AtHEXO3. However, the transmembrane prediction for AtHEXO3 might not be real as it also has a predicted signal peptide for secretion.

Altogether, five full-length HEXO ORFs were retrieved from *N. benthamiana* cDNA (Supplementary Table 2) and could be screened for their functional characterization on complex LDN-carrying N-glycans (Figure 1A).

### Subcellular Localization of *N. benthamiana* HEXOs

**FIGURE 2:**
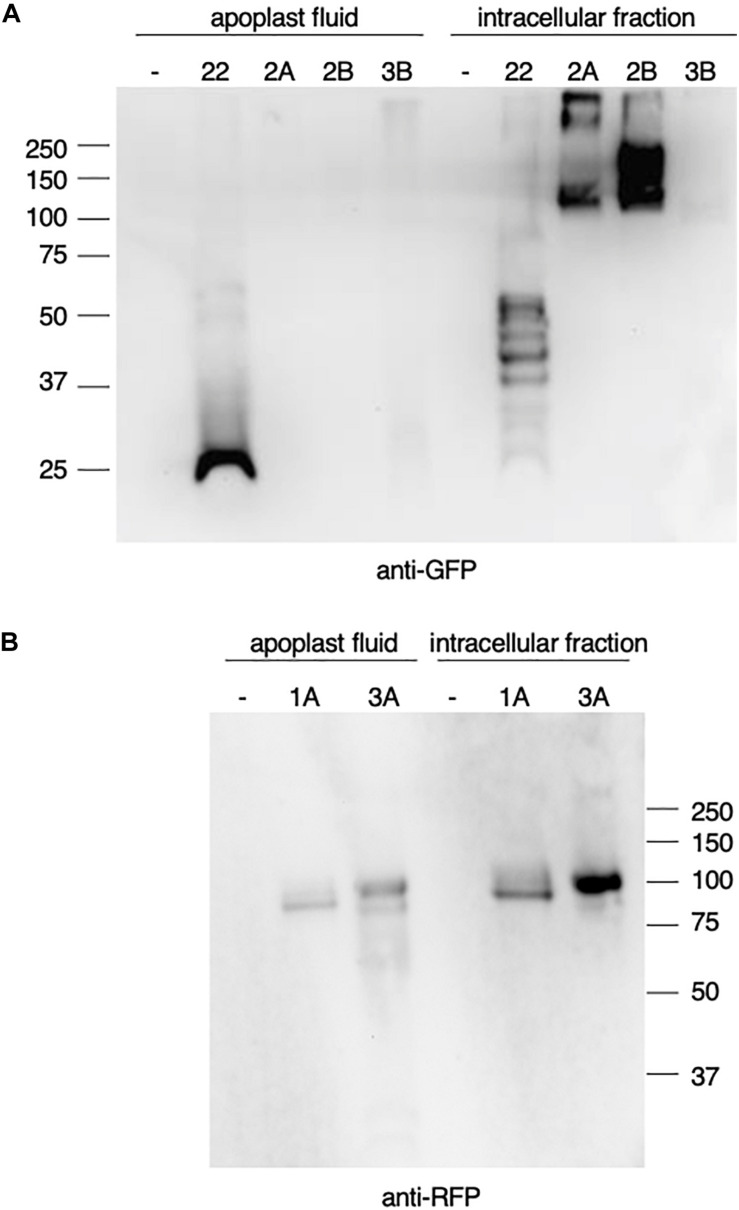
Expression of *Nicotiana benthamiana* beta-hexosaminidases (HEXOs). GFP- or RFP-tagged HEXOs or IL-22-GFP (used as apoplast control protein) were transiently expressed in *N. benthamiana* leaves. As a negative control (–) p19 was used. Leaves were harvested 3 days post infiltration and analyzed by western blot. **(A)** anti-GFP western blot under reducing conditions of 10 μg total soluble protein of apoplast fluids or intracellular fraction upon expression of IL-22-GFP (22), HEXO2A (2A), HEXO2B (2B), or HEXO3B (3B). **(B)** anti-RFP western blot under reducing conditions of 10 μg total soluble protein of apoplast fluids or intracellular fraction upon expression of HEXO1A (1A) or HEXO3A (3A).

Prior to the functional characterization of the HEXO genes we expressed the enzymes transiently in plant leaves and analyzed their expression and subcellular localization. GFP- and RFP-tagged HEXOs (Figure 1B) were expressed in *N. benthamiana* leaves after which their distribution in the apoplast or in the remaining intracellular fraction was analyzed by western blot. GFP-tagged human IL-22 was taken along as an apoplastic control protein, which results in the detection of free GFP at ± 25 kDa as a result of proteolytic cleavage in the apoplast and multiple glycosylated variants of the fusion protein (±45–55 kDa and smaller cleavage products) in the intracellular fraction (Figure 2A).

Analysis of the subcellular distribution of *N. benthamiana* HEXOs revealed that HEXO1A-RFP was clearly detected at ±100 kDa in the intracellular fraction, but could also be retrieved from the apoplast (Figure 2B). The latter was quite unexpected because HEXO1A is reported to be vacuolar protein (Shin et al., 2017). HEXO2A-GFP and HEXO2B-GFP were clearly detected in the intracellular fraction (>100 kDa) and tend to form dimers and/or multimers (Figure 2A). No signal was detected for HEXO2 fusion proteins in the apoplast, which suggests that HEXO2A and HEXO2B are not secreted. As expected, our western blot analysis also shows that HEXO3A-RFP can be retrieved from the apoplast (Figure 2B). Additionally, HEXO3A-RFP was clearly detected at ±100 kDa in the remaining intracellular fraction. On the other hand, HEXO3B-GFP seemed to be expressed at much lower levels, since only faint signals were detected in the intracellular fraction and apoplast at ±100 kDa (Figure 2A).

**FIGURE 3:**
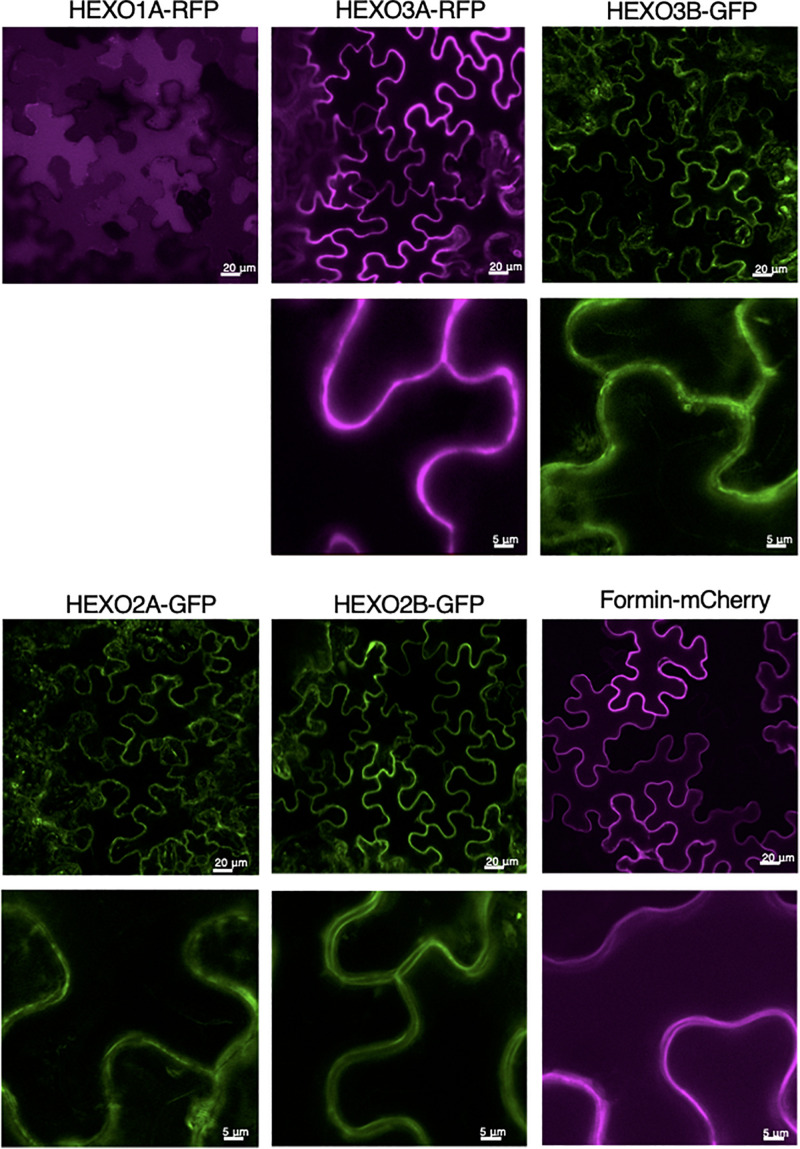
Subcellular localization of *Nicotiana benthamiana* HEXOs. GFP- or RFP-tagged HEXOs or formin-mCherry (used as plasma membrane marker) were transiently expressed in *N. benthamiana* leaves. Whole mount confocal microscopy pictures were taken at 3 days post infiltration. For each construct overview pictures are given (scale bar of 20 μm) and a picture at higher magnification underneath (scale bar 5 μm).

To examine HEXO subcellular localization in more detail, we imaged the fluorescently tagged HEXOs in leaf epidermal cells with confocal microscopy. As expected, HEXO1A-RFP was clearly distributed in the vacuole (Figure 3). Both HEXO2A-GFP and HEXO2B-GFP give a clear outline of the epidermal cells, although other subcellular structures/organelles are visible as well. Still, the outline of the epidermal cells is more distinct upon the expression of HEXO2B-GFP. At higher magnification, we observed two parallel outlines of the epidermal cells for HEXO2B-GFP with no fluorescence between the cells. Based on the fact that HEXO2B-GFP could not be retrieved from the apoplast (Figure 2A) and has a predicted transmembrane domain, we hypothesized that HEXO2B localizes in the plasma membrane. For this reason, we examined the subcellular localization of mCherry-fused formin upon transient expression, which is a membrane-bound protein from Arabidopsis. Formin-mCherry clearly delineates the border of the epidermal cells and, at higher magnification, looks similar to HEXO2B-GFP. We therefore suggest that HEXO2B is a membrane bound enzyme. Finally, HEXO3A-RFP clearly marks the boundary of epidermal cells, which is indicative for its apoplastic localization as previously reported (Shin et al., 2017). In comparison, HEXO3B-GFP localization was not as distinctive as seen for HEXO3A-RFP. The outline of the epidermal cells is less clear and other subcellular structures/organelles are visible as well.

### HEXO1A and HEXO3A Are Responsible for the Formation of Paucimannosidic Glycans

**FIGURE 4:**
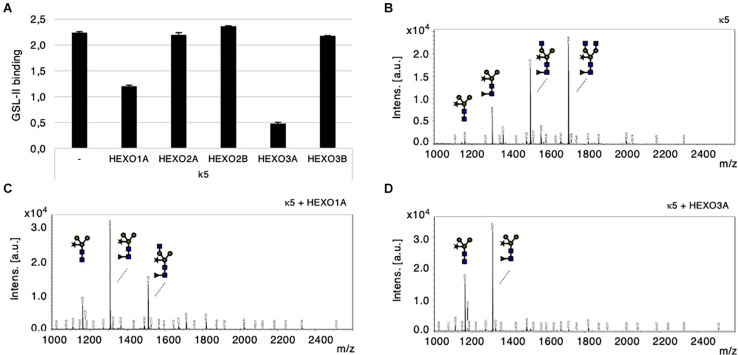
Cleavage of terminal GlcNAc residues by *N. benthamiana* HEXOs. Kappa-5 was co-expressed with p19 and different HEXO genes in wild-type plants. After isolation of the apoplast fluid (at 6 dpi) the glycan composition of kappa-5 was analyzed. **(A)** *Griffonia simplicifolia* lectin II (GSL-II) binding assay for detection of terminal GlcNAc. Lectin binding is measured as absorbance at 450 nm. **(B–D)** Kappa-5 was purified from the apoplast fluid and the N-glycan composition was analyzed by MALDI-TOF-MS. MS profiles are given for N-glycans of wild-type kappa-5 **(B)** or upon co-expression of HEXO1A **(C)** or HEXO3A **(D)**. m/z refers to mass-to-charge ratio.

For functional characterization of *N. benthamiana* HEXOs, we first co-expressed individual HEXO genes with kappa-5 as a carrier protein for complex N-glycans with terminal GlcNAc residues (GnGnXF^3^; Wilbers et al., 2017). Kappa-5 was then extracted from the apoplast and analyzed with the GSL-II lectin, which binds terminal GlcNAc residues. As expected, GSL-II binds strongly to the N-glycans of kappa-5 expressed in wild-type plants (Figure 4A). Upon co-expression of individual HEXOs, the signal for GSL-II binding dropped for HEXO1A and to an even stronger extent for HEXO3A. Co-expression of HEXO2A, HEXO2B, or HEXO3B did not affect the binding of GSL-II to the N-glycans on kappa-5.

Mass spectrometric (MS) analysis on PNGase A released N-glycans from purified kappa-5 was then performed to confirm the cleavage of terminal GlcNAc residues. PNGase A was used because it releases all N-glycans from glycopeptides and therefore includes core α1,3-fucosylated N-glycans. As expected, the major N-glycan found on kappa-5 produced in wild-type plants was the GnGnXF^3^ structure (Figure 4B). Two other peaks are visible as well and represent N-glycans of kappa-5 that are processed by endogenous HEXOs. Upon co-expression of HEXO1A, only a small fraction of the N-glycans carried a single terminal GlcNAc residue and the majority was trimmed down to paucimannosidic (MMXF^3^) N-glycans (Figure 4C). Co-expression of HEXO3A resulted in a complete lack of GnGnXF^3^ glycans on kappa-5 (Figure 4D), which coincides with the stronger drop in GSL-II binding (Figure 4A). Altogether, we conclude that only HEXO1A and HEXO3A are responsible for the formation of paucimannosidic N-glycans.

### HEXO2B and HEXO3A Are Responsible for Trimming GalNAc Residues of N-glycans

**FIGURE 5:**
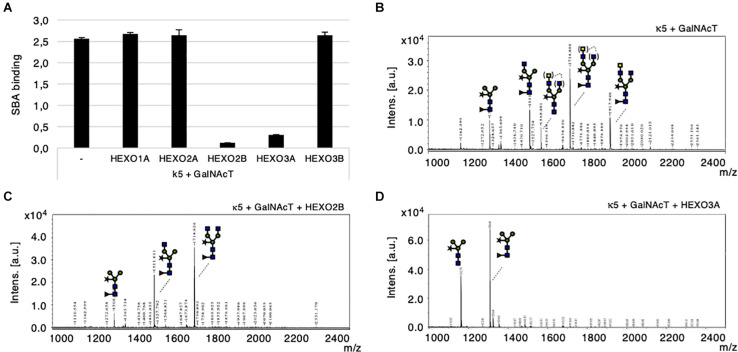
Cleavage of engineered LDN motifs by *N. benthamiana* HEXOs. Kappa-5 was co-expressed with p19, CeGalNAcT and different HEXO genes in wild-type plants. After isolation of the apoplast fluid (at 6 dpi) the presence of the LDN motif on kappa-5 N-glycans was analyzed. **(A)** Soybean agglutinin (SBA) binding assay for detection of terminal GalNAc. Lectin binding is measured as absorbance at 450 nm. **(B–D)** Kappa-5 was purified from the apoplast fluid and the N-glycan composition was analyzed by MALDI-TOF-MS. MS profiles are given for N-glycans of kappa-5 upon co-expression of GalNAcT only **(B)**, or combined with co-expression of HEXO2B **(C)**, or HEXO3A **(D)**. When a MS peak represents multiple N-glycan structures of identical mass, the possible positions of these sugar residues on the N-glycan are indicated between brackets. m/z refers to mass-to-charge ratio.

To investigate whether HEXOs from *N. benthamiana* are able to cleave GalNAc residues from N-glycans, we co-expressed kappa-5 with GalNAcT from *C. elegans* (CeGalNAcT) in wild-type *N. benthamiana* plants in order to synthesize β1,4-GalNAc extended N-glycan branches (LDN-glycans). Individual HEXOs were then co-expressed to screen for cleavage activity toward LDN-glycans on kappa-5. Kappa-5 was extracted from the leaf apoplast and analyzed for the presence of LDN-glycans with the SBA lectin, which binds terminal GalNAc residues. As expected, SBA bound strongly to the N-glycans of kappa-5 upon co-expression of CeGalNAcT (Figure 5A). Interestingly, the binding of SBA was strongly reduced upon co-expression of HEXO2B or HEXO3A, which indicates their ability to cleave LDN-glycans.

Mass spectrometric analysis on PNGase A released N-glycans from purified kappa-5 was then performed to confirm the cleavage of terminal GalNAc residues. Figure 5B illustrates a N-glycan profile for kappa-5 N-glycans upon engineering of LDN as was previously published (Wilbers et al., 2017). Enzymatic treatment with a specific β-*N*-acetyl-glucosaminidase was included to confirm the proportion of LDN in each sample (Supplementary Figure 4D). Upon co-expression of HEXO2B or HEXO3A we did no longer detect LDN motifs (Figures 5C,D, respectively). These data also reveal distinct substrate specificities for *N. benthamiana* HEXOs, where HEXO2B solely cleaves of GalNAc residues and HEXO3A trims down N-glycans completely to paucimannosidic structures. Furthermore, we confirmed that HEXO1A does not cleave GalNAc (Supplementary Figure 5) and therefore seems to be specific for GlcNAc residues on N-glycans.

Altogether, we conclude that HEXO2B and HEXO3A are responsible for trimming down LDN-glycans of kappa-5 in *N. benthamiana*.

### Simultaneous Knockdown of HEXO2 and HEXO3 by RNAi Is Not Sufficient to Improve the Synthesis of LDN-Glycans

**FIGURE 6:**
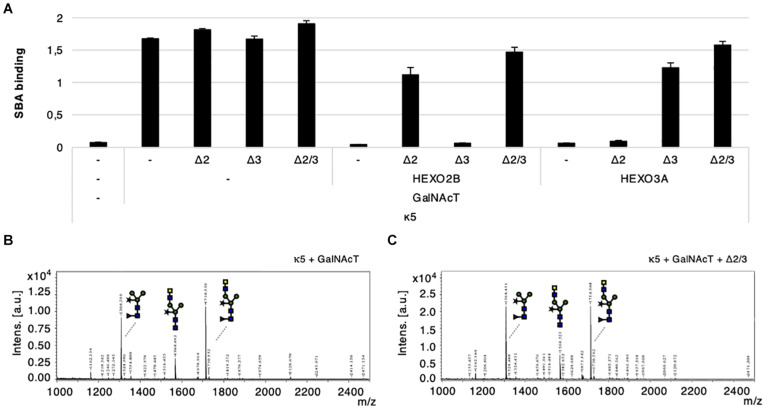
Transient silencing of HEXO2 and HEXO3. Kappa-5 was co-expressed with CeGalNAcT and constructs targeting HEXO2 (Δ2), HEXO3 (Δ3), or both (Δ2/3). The samples are grouped in three blocks depending on whether a HEXO variant (2B or 3A) was co-expressed. Control samples where a particular construct is not co-infiltrated is indicated as well (–). After isolation of the apoplast fluid (at 5 dpi) the presence of the LDN motif on kappa-5 N-glycans was analyzed. **(A)** Soybean agglutinin (SBA) binding assay for detection of terminal GalNAc. Lectin binding is measured as absorbance at 450 nm. **(B,C)** Kappa-5 was purified from the apoplast fluid and the N-glycan composition was analyzed by MALDI-TOF-MS. MS profiles are given for glucosaminidase treated N-glycans of kappa-5 upon co-expression of GalNAcT only **(B)** or combined with the hairpin construct targeting HEXO2 and HEXO3 (Δ2/Δ3; **C**). m/z refers to mass-to-charge ratio.

In order to optimize the glyco-engineering of N-glycans carrying the helminth glycan motif LDN, we attempted to transiently silence HEXO2 and/or HEXO3 genes with hairpin constructs. We co-expressed kappa-5, CeGalNAcT and different hairpin constructs [targeting HEXO2 (Δ2), HEXO3 (Δ3), or both (Δ2/3)] in wild-type plants. In addition, we overexpressed HEXO2B or HEXO3A in order to check for silencing specificity. Kappa-5 was extracted from the leaf apoplast and screened for the presence of LDN motifs with the SBA lectin. As expected, over-expression of either HEXO2B or HEXO3A greatly reduces the binding of SBA to the N-glycans of kappa-5 after LDN engineering. The activity of both enzymes was efficiently neutralized by co-expression of different hairpin constructs (Figure 6A). This experiment reveals that the hairpin constructs targeting a single HEXO gene are highly specific since they only block the activity of their respective targets. Additionally, the hairpin construct targeting both HEXO2 and HEXO3 seems to block the activity of over-expressed HEXOs most efficiently.

In the setting of LDN-glycan engineering (in the absence of HEXO over-expression), it appears that SBA binding to kappa-5 LDN-glycans increases slightly upon co-expression of the Δ2 and Δ2/Δ3 hairpin constructs (Figure 6A). This could suggest that HEXO2B plays a larger role in cleaving GalNAc residues than HEXO3A. However, when analyzing the presence of LDN on kappa-5 N-glycans by MS we did not observe an improvement of LDN synthesis (Figures 6B,C). The MS profiles are given for kappa5 LDN-glycans with or without co-expression the Δ2/Δ3 hairpin construct (after β-*N*-acetyl-glucosaminidase treatment). Both MS profiles showed a similar LDN content on kappa-5 and show that simultaneous knock-down of HEXO2 and HEXO3 is not sufficient to improve LDN-glycan synthesis.

## Discussion

*Nicotiana benthamiana* has become an established expression system for recombinant proteins with therapeutic value (Stoger et al., 2014). In addition, *N. benthamiana* offers a highly versatile expression platform for the production of glycoproteins when it comes to engineering tailor-made human N-glycans (Bosch et al., 2013; Montero-Morales and Steinkellner, 2018) and more recently for N-glycans of parasitic helminths (Wilbers et al., 2017). Upon expression in plants, undesired processing of N-glycans on glycoproteins can occur by β-hexosamindases (HEXOs) along the secretory pathway. We have previously hypothesized that both GlcNAc and GalNAc residues of LDN-glycans can be targeted by HEXOs in *N. benthamiana*. In this study, we investigated if HEXOs of *N. benthamiana* are responsible for the trimming of GalNAc residues from LDN-glycans on apoplast glycoproteins. Therefore, we cloned novel HEXO ORFs from *N. benthamiana* and expressed them transiently alongside kappa-5 as a carrier glycoprotein for engineered LDN-glycans. With this approach we were able to identify HEXO2 and HEXO3 as enzymes capable of trimming down LDN-glycans, where HEXO2 has strict specificity for GalNAc residues and HEXO3 cleaves both GlcNAc and GalNAc.

To identify HEXO enzymes from *N. benthamiana* responsible for the cleavage of LDN-glycans, we first amplified HEXO1, HEXO2, and HEXO3 ORFs to get a complete overview of tobacco orthologs in this enzyme family. Because of the allotetraploid nature of the *N. benthamiana* genome (Bombarely et al., 2012) we expected to find two orthologous ORFs for each HEXO variant described in *A. thaliana* (AtHEXO1-3). Previous work by Shin et al. (2017) already described two HEXO ORFs, which are referred to as HEXO1A and HEXO3A in our study. In addition to HEXO1A and HEXO3A, we retrieved two novel ORFs for HEXO2 (HEXO2A and HEXO2B) and an additional HEXO3 ORF (HEXO3B). A likely reason why a HEXO1B ORF could not be retrieved is that the coding region for the first 143 amino acids is missing in the genomic context of this ortholog. The DNA encoding these 143 amino acids can be traced back to a contig and could be located on a different position in the genome due to a recombination event. This suggests that after duplication of the genome only the HEXO1A gene has remained intact and is sufficient to perform its activity. Similarly, our study reveals that for HEXO2 and HEXO3 orthologs, only HEXO2B and HEXO3A encode active enzymes. Therefore, duplicated HEXO genes seem to have lost their function during the course of evolution.

β-hexosaminidase activity in plants is largely restricted by their localization and leads to the formation of paucimannosidic (MMXF^3^) N-glycans in either the vacuole by HEXO1 or in the apoplast by HEXO3 (Strasser et al., 2007; Liebminger et al., 2011; Shin et al., 2017). Even though HEXO1 activity is normally restricted to the vacuole we observed that the processing of kappa-5 N-glycans to paucimannosidic structures is almost complete upon over-expression of HEXO1A. HEXOs travel through the Golgi to reach their destination (Strasser et al., 2007), and it has been shown that both HEXO1A and HEXO3A have Golgi-processed N-glycans (Shin et al., 2017). In the case of HEXO1A, it would therefore be possible that GlcNAc cleavage of N-glycans already occurs prior to the secretion of kappa-5. However, upon over-expression, we also detect a significant amount of HEXO1A in the apoplast. HEXO1A could leak into the apoplast when there is simply too much enzyme produced and transport to vacuole becomes overloaded. In addition to GlcNAc processing, the N-glycans of kappa-5 are also cleaved by HEXOs upon engineering of LDN-glycans. Our study shows that HEXO2B and HEXO3A are able to cleave GalNc and this coincides with their localization. HEXO2B likely acts on the N-glycans of kappa-5 as a membrane-bound enzyme with an apoplastically localized catalytic domain. As mentioned, HEXO3A travels through the secretory pathway to apoplast, where it can act on the LDN-glycans of kappa-5 as well. The processing of kappa-5 N-glycans could therefore occur along the entire secretory pathway.

Activity of HEXOs toward N-glycans on different glycoproteins can vary, which is illustrated previously by the production of human IL-22, venom allergen-like protein 1 (VAL-1) from *Brugia malayi*, VAL-4 from *Heligmosomoides polygyrus* and *S. mansoni* secreted egg antigens omega-1 and kappa-5 in *N. benthamiana* (Wilbers et al., 2016, 2017; Asojo et al., 2018; Darwiche et al., 2018). All these proteins have previously been expressed and purified from wild-type *N. benthamiana* plants using the same apoplast expression strategy. Yet, even though these glycoproteins were isolated from the same subcellular compartment they all carry N-glycans with a different composition of HEXO processed N-glycans (MMXF^3^, GnMXF^3^, or GnGnXF^3^). For example, the N-glycans of omega-1 are completely paucimannosidic (MMXF^3^) upon transient expression in wild-type *N. benthamiana*, whereas the N-glycans on kappa-5 seem to be resistant to HEXO activity as the majority of these N-glycans carry terminal GlcNAc residues (GnGnXF^3^; Wilbers et al., 2017). In this study, we now also demonstrate that transient over-expression of HEXO1A or HEXO3A results in the (almost) complete processing of seemingly “HEXO-resistant” N-glycans on kappa-5 to paucimannosidic glycans. Processing of N-glycans by HEXOs is therefore not only dependent on N-glycan accessibility, but also on the expression level of HEXOs themselves.

Dual specificity of HEXOs toward GlcNAc and GalNAc residues lies within their name, but so far plant HEXOs have been shown to have a strong preference for chemically synthesized GlcNAc substrates (Strasser et al., 2007). In plants, HEXO1 and HEXO3 contribute equally to the formation of paucimannosidic N-glycans (Liebminger et al., 2011; Shin et al., 2017). HEXO1 constitutes 90% of activity for soluble HEXOs, and HEXO3 activity is mainly found in the insoluble fraction (Liebminger et al., 2011). Furthermore, there does not seem to be a preference for processing of GlcNAc residues from different N-glycan arms in plants (Gutternigg et al., 2007; Strasser et al., 2007), which is further illustrated in our study by the complete processing of kappa-5 N-glycans after over-expressing tobacco HEXO1A or HEXO3A. This is in contrast to some HEXOs from invertebrate animals, like HEX-2 and HEX-3 from *C. elegans* and FDL HEXO from *Drosophila melanogaster*. They only process the GlcNAc residue from the α1,6-arm of N-glycans, thereby generating GnM structures (Gutternigg et al., 2007). Although the processing of GlcNAc residues from N-glycans is well described for HEXOs in plants, no reports exist on the processing of GalNAc from N-glycans by these enzymes. Our study is the first to investigate the ability of plant HEXOs to process complex LDN-glycans and reveals that tobacco HEXO2B and HEXO3A are able to process LDN, where HEXO2 has strict specificity for GalNAc residues. A similar observation has been made for HEXOs from *C. elegans* and their ability to process LDN-glycans (Léonard et al., 2006; Gutternigg et al., 2007; Dragosits et al., 2015). This work demonstrated that HEX-2 and HEX-3 cleave both GlcNAc and GalNAc from LDN-glycans, whereas HEX-4 and HEX-5 strictly cleave GalNAc residues. In contrast, *D. melanogaster* FDL shows no activity toward LDN-glycans (Dragosits et al., 2015). Regarding substrate specificity for GlcNAc and/or GalNAc residues on N-glycans, tobacco HEXO2B is very similar to HEX-4 and HEX-5 with strict galactosaminidase activity. On the other hand, HEXO3A activity resembles the activity of HEX-2 and HEX-3 and is a true hexosaminidase when it comes to processing N-glycans. The inability of tobacco HEXO1A to process LDN-glycans makes it similar to FDL from *D. melanogaster* with strict glucosaminidase activity toward N-glycan substrates (Léonard et al., 2006; Dragosits et al., 2015).

The ability of plant HEXOs to cleave the monosaccharide GalNAc is quite remarkable, since to our knowledge no endogenous polysaccharide substrates containing GalNAc exist in plants. So far, incorporation of GalNAc into N- or O-glycans has only been documented for glyco-engineered plants (Castilho et al., 2012; Yang et al., 2012; Wilbers et al., 2017). The ability of HEXO2B and HEXO3A to process non-endogenous glycans hints toward a role in defense against invading organisms that do have LDN-glycans. The interplay of pathogen/parasite-associated glycans with plant immunity is a fairly unexplored research area, which is in contrast to the strong focus on glycans in research regarding animal parasitic worms (Hokke and Yazdanbakhsh, 2005; Tundup et al., 2012). A role for HEXOs in plant defense responses is further supported by the chitinolytic activity of several plant HEXOs (Gutternigg et al., 2007; Strasser et al., 2007; Ryšlavá et al., 2014; Hyskova, 2015). Where the chitinolytic activity of HEXOs in other organisms could play a role in the turnover of chitin in the cell wall (fungi), exoskeleton (insects), or cuticle (nematodes), plants might employ these enzymes to protect themselves against the constant threat of fungi, insects, and nematodes. These observations could open up a new research area where the role of pathogen- or parasite-associated glycans in the host–parasite interface would be the target of investigation.

The ultimate goal of this study was to improve the engineering of LDN-glycans in plants by identifying the HEXO orthologs of *N. benthamiana* responsible for GalNAc cleavage. After identifying HEXO2 and HEXO3 as candidate enzymes, we employed RNAi-mediated knock-down of these genes to block the undesired processing of LDN-glycans. Unfortunately, in our experimental set-up, we were not able to improve the synthesis of LDN-glycans after simultaneous knock-down of HEXO2 and HEXO3. This was somewhat unexpected, since the Δ3 hairpin construct was previously used to effectively reduce the formation of paucimannosidic N-glycans on α1-antitrypsin (A1AT; Shin et al., 2017). However, the efficacy of this hairpin construct is dependent on the glycopeptide of A1AT that is analyzed and seems to be lower when analyzing the N-glycan composition of total apoplast proteins. In this set-up, it would still be possible that HEXO1 (instead of HEXO3) processes GlcNAc residues from N-glycans of glycoproteins before they reach the apoplast (as discussed before) or that transient knock-down is simply not sufficient to completely abolish HEXO3 activity in the apoplast. As the processing of N-glycans by HEXOs seems to be dependent on the target protein, N-glycan accessibility, and HEXO expression, it would be best to completely knock-out the *hexo* genes responsible for undesired N-glycan processing.

In conclusion, we reveal a novel role for plant HEXOs in the processing of N-glycans on apoplastic glycoproteins. Furthermore, this is the first report to describe a biochemical role for HEXO2 in an *in vivo* setting. The identification of HEXO2 and HEXO3 as major targets for LDN cleavage could enable an effective strategy to reduce undesired processing of these N-glycans post secretion. Effective knockout of HEXO enzymes by genome editing approaches will optimize the plant-based production of therapeutically relevant glycoproteins with tailor-made helminth N-glycans in plants.

## Data Availability Statement

The original contributions presented in the study are included in the article/Supplementary Material, further inquiries can be directed to the corresponding author/s.

## Author Contributions

NA, KN, NG, and OS were involved in the design and/or execution of confocal experiments. NA, KN, SD, NG, KV, AS, and RW were involved in the design and/or execution of experiments regarding cloning of HEXO genes, plant-based expression, protein purification, and glyco-engineering. D-LN and CH were involved in the glycan structural analysis. RS shared valuable construct for this study. RW supervised the project. NA and RW wrote the manuscript with input from all other authors. All authors contributed to the article and approved the submitted version.

## Conflict of Interest

The authors declare that the research was conducted in the absence of any commercial or financial relationships that could be construed as a potential conflict of interest.

## Supplementary Material

Supplementary Figure 1 Amino acid sequence alignment (Clustal Omega) of *A. thaliana* HEXO1, predicted open reading frames (ORFs) of HEXO1 orthologs from the Sol Genomics Network draft genome for *N. benthamiana* (v1.0.1) and retrieved HEXO1 ORFs from Shin et al. (2017). For one predicted ORF, the sequences of Niben101Ctg15557g00001.1 (highlighted in orange) and Niben101Scf10015g07014.1 were combined.

Supplementary Figure 2 Amino acid sequence alignment (Clustal Omega) of *A. thaliana* HEXO2, predicted open reading frames (ORFs) of HEXO2 orthologs from the Sol Genomics Network draft genome for *N. benthamiana* (v1.0.1) and retrieved HEXO2 ORFs in this study. For one predicted ORF we combined the sequences of Niben101Scf15928g00003.1 and Niben101Scf01151g00003.1.

Supplementary Figure 3 Amino acid sequence alignment (Clustal Omega) of *A. thaliana* HEXO3, predicted open reading frames (ORFs) of HEXO3 orthologs from the Sol Genomics Network draft genome for *N. benthamiana* (v1.0.1) and retrieved HEXO3 ORFs from Shin et al. (2017) and this study.

Supplementary Figure 4 HEXO2B substrate specificity. Kappa-5 was co-expressed with p19 and combinations of HEXO2B and/or CeGalNAcT in wild-type plants. Kappa-5 was purified from the apoplast fluid (at 6 dpi) and the N-glycan composition of kappa-5 was analyzed MALDI-TOF-MS. MS profiles are given for N-glycans of wild-type kappa-5 **(a)**, kappa-5 with HEXO2B **(b)**, kappa-5 with CeGalNAcT **(c)**, and kappa-5 with CeGalNAcT and HEXO2B **(d)**. To distinguish terminal GlcNAc and GalNAc residues we treated the N-glycans with glucosaminidase for which the substrate specificity is indicated in the dashed box. When a MS peak represents multiple N-glycan structures of identical mass, the possible positions of these sugar residues on the N-glycan are indicated between brackets.

Supplementary Figure 5 HEXO1A substrate specificity. Kappa-5 was co-expressed with p19, CeGalNAcT and with/without HEXO1A in wild-type plants. Kappa-5 was purified from the apoplast fluid (at 6 dpi) and the N-glycan composition of kappa-5 was analyzed MALDI-TOF-MS. MS profiles are given for N-glycans of kappa-5 with CeGalNAcT **(a)** and kappa-5 with CeGalNAcT and HEXO1A **(b)**. To distinguish terminal GlcNAc and GalNAc residues we treated the N-glycans with glucosaminidase for which the substrate specificity is indicated in the dashed box. When a MS peak represents multiple N-glycan structures of identical mass, the possible positions of these sugar residues on the N-glycan are indicated between brackets.

Supplementary Figure 6 Nucleotide sequence alignment (Clustal Omega) of HEXO2A, HEXO2B, and the target region of the Δ2 hairpin construct.

Supplementary Figure 7 Nucleotide sequence alignment (Clustal Omega) of HEXO3A, HEXO3B, and the target region of the Δ3 hairpin construct.

Supplementary Table 1 Primer overview. This table gives an overview of all the primers used in this study to perform 3′ RACE for HEXO2 orthologs and amplification of full-length HEXO open reading frames. Restriction sites used for subcloning are underlined. Capital letters refer to gene specific sequences.

Supplementary Table 2 Overview of β-Hexosaminidases. This table gives an overview of HEXO sequences from *Arabidopsis thaliana* and *Nicotiana benthamiana* reported in literature and from this study. For each HEXO variant we provide the accession number, homology between the two plant species, information on whether the gene encodes a functional protein, size of the encoded protein, presence of a signal peptide for secretion (SP) and/or transmembrane domain (TMD), the cellular localisation, and literature citations. Sequence homology between Arabidopsis and Nicotiana orthologs was determined on amino acid level by using Clustal Omega alignments. The signal peptide was predicted by the SignalP server (v5) and the transmembrane domain was predicted by the TMHMM server v2.0. Y: yes; N: no.
