# Supplementary material for: β-Hexosaminidases Along the Secretory Pathway of Nicotiana benthamiana Have Distinct Specificities Toward Engineered Helminth N-Glycans on Recombinant Glycoproteins
Source: Front Plant Sci. 2021 Mar 17;12:638454. doi: 10.3389/fpls.2021.638454 (PMC8010188; doi:10.3389/fpls.2021.638454)
Supplement: Supplementary Figure 1 — Amino acid sequence alignment (Clustal Omega) of A. thaliana HEXO1, predicted open reading frames (ORFs) of HEXO1 orthologs from the Sol Genomics Network draft genome for N. benthamiana (v1.0.1) and retrieved HEXO1 ORFs from Shin et al. (2017). For one predicted ORF, the sequences of Niben101Ctg15557g00001.1 (highlighted in orange) and Niben101Scf10015g07014.1 were combined. [file Data_Sheet_1.pdf]

Supplemental information belonging to the manuscript:

β-Hexosaminidases along the secretory pathway of *Nicotiana benthamiana* have distinct specificities towards engineered helminth N-glycans on recombinant glycoproteins

Nicolò Alvisi[1], Kim van Noort[1], Sarlita Dwiani[1], Nathan Geschiere[1], Octavina Sukarta[1], Koen Varossieau[1], Dieu-Linh Nguyen[2], Richard Strasser[3], Cornelis H. Hokke[2], Arjen Schots[1] and Ruud H. P. Wilbers[1*]

* corresponding author

1 Laboratory of Nematology, Plant Sciences Group, Wageningen University and Research, Droevendaalsesteeg 1, 6708 PB, Wageningen, The Netherlands

2 Department of Parasitology, Leiden University Medical Center, Albinusdreef 2333 ZA, Leiden, The Netherlands

3 Department of Applied Genetics and Cell Biology, University of Natural Resources and Life Sciences, Vienna, Austria

*Corresponding author: Ruud H.P. Wilbers, P.O. Box 8123, 6700 ES Wageningen, The Netherlands, phone: +31(0)317485261, fax: +31(0)317484254, e-mail: ruud.wilbers@wur.nl

Table S1. Primer overview

This table gives an overview of all the primers used in this study to perform 3' RACE for HEXO2 orthologs and amplification of full-length HEXO open reading frames. Restriction sites used for subcloning are underlined. Capital letters refer to gene specific sequences.

Primer name	Sequence (5' – 3')	Characteristics (underlined)
HEXO1A-fw	cctcatgagcATGTCCTCAAATTCCCAATGTC	BspHI site for subcloning into pHYG
HEXO1A-rv	ggtgtacaTTATTGTTCATAGCATGATCCTGG	BsrGI site for subcloning into pHYG
HEXO2A-fw	cctcatgagcATGGCTTACTTCATTTCATTTCTCTCC	BspHI site for subcloning into pHYG
HEXO2A-rv	ggggtaccTCAGCTGGTAAATGGATGAACTGTG	KpnI site for subcloning into pHYG
HEXO2B-fw	cctcatgagcATGGCTTACTTCAACTTCAAAG	BspHI site for subcloning into pHYG
HEXO2B-rv	ggggtaccTCAGCTGGTAAATGGATGAAC	KpnI site for subcloning into pHYG
HEXO3A-fw	cctcatgagcATGGGGAAGTTAGGATTCCG	BspHI site for subcloning into pHYG
HEXO3B-fw	ccccATGGGGAAGTTAGGAGTGCA	NcoI site for subcloning into pHYG
HEXO3-rv	ggggtaccTTATTGCTGATAGCAAGAACCTG	KpnI site for subcloning into pHYG
Oligo(dT)-AP	ggccacgcgtcgactagtacTTTTTTTTTTTTTTTTT	Adaptor sequence for 3' RACE
Adaptor primer	ggccacgcgtcgactagtac	Reverse primer for 3' RACE of HEXO2
HEXO2-RACE-fw	TCCTTCGGAGCCGGAGCTC	Forward primer for 3' RACE of HEXO2

Table S2. Overview of β-Hexosaminidases

This table gives an overview of HEXO sequences from *Arabidopsis thaliana* and *Nicotiana benthamiana* reported in literature and from this study. For each HEXO variant we provide the accession number, homology between the two plant species, information on whether the gene encodes a functional protein, size of the encoded protein, presence of a signal peptide for secretion (SP) and/or transmembrane domain (TMD), the cellular localisation, and literature citations. Sequence homology between Arabidopsis and Nicotiana orthologs was determined on amino acid level by using Clustal Omega alignments. The signal peptide was predicted by the SignalP server (v5) and the transmembrane domain was predicted by the TMHMM server v2.0. Y: yes; N: no.

	Accession	Protein coding / Active	Homology with Arabidopsis (%)	Protein Length (aa)	SP (aa)	TMD (aa)	Localisation	Citation
A. thaliana								
HEXO1	At3g55260	Y / Y	-	541	1-20		vacuole	[8, 14]
HEXO2	At1g05590	Y / Y	-	580	1-28	7-29	plasma membrane, apoplast	[8, 14]
HEXO3	At1g65590	Y / Y	-	535	1-24	7-26	plasma membrane, apoplast	[8, 14]
N. benthamiana								
HEXO1A	Niben101Scf03794g01004.1	Y / Y	71.51	541	1-24	-	vacuole	[15], this manuscript
HEXO1B	Niben101Scf10015g07014.1	N	-	-	-	-	-	this manuscript
HEXO2A	Niben101Scf09360g01009	Y / N	59.54	548	-	21-43	plasma membrane, apoplast	this manuscript
HEXO2B	Niben101Scf15928g00003.1<br>Niben101Scf01151g00003.1	Y / Y	63.53	602	-	28-50	-	this manuscript
HEXO3A	Niben101Scf02017g00001	Y / Y	68.30	530	1-24	-	apoplast	[15], this manuscript
HEXO3B	Niben101Scf02405g02003	Y / N	69.13	530	1-24	-	-	this manuscript

```
AtHEXO1                 MSTN-------LLRLILLFI---TLSITSSL-STPSPADSPPYLWPLPAEFSFGNETLSV    49
HEXO1A                  MSSNSQCLLKTLFFLLFIFPLVNARSIKSTLHKTTELDESLTYLWPLPSQFTFGNDTLTV    60
101-03794               MSSNSQCLLKTLFFLLFIFPLVNARSIKSTLHKTTELDESLTYLWPLPSQFTFGNDTLTV    60
Ctg15557/101-10015      MSSNSQFLLKTLSFLLLILPLVNSRSTKSTLHKTTELDESLTYLWPLPSQFTFANDTLTV    60
                        **:*       *  *::::    : * .*:*  .* . :*  ******::*:*.*:**:*

AtHEXO1                 DPTVTLIVAGNGGGSLIIRAAFDRYMGIIFKHASGRGSLLSRIRFLKMVEYDITSLKIVV    109
HEXO1A                  DPNLSIVFTGNGGGSVIVKEAFERYKKIIFKHGSKSGDF-----------FDVTQLTVIV    109
101-03794               DPNLSIVFTGNGGGSVIVKEAFERYKKIIFKHGSKSGDF-----------FDVTQLTVIV    109
Ctg15557/101-10015      DPNLSIVFTGNGGESVIVKQAFERYKKIIFKHGSKSGEF-----------FDVTQLTVIV    109
                        **.::::.:**** *:*:: **:**  *****.*  *.:           :*:*.*.::*

AtHEXO1                 HSDSEELQLGVDESYTLMVSKKNEQSIVGAATIEANTVYGALRGLETFSQLCAFDYITKS    169
HEXO1A                  HSDNDELQLGVDESYSLLVTKSNEHSIIGEVSIEANSIYGALRGLETMSQLCIFDYGVKT    169
101-03794               HSDNDELQLGVDESYSLLVTKSNEHSIIGEVSIEANSIYGALRGLETMSQLCIFDYGVKT    169
Ctg15557/101-10015      HSDNDELQLGVDESYSLLVTKSNKHSIIGEVSIEANSIYGALRGLETMSQLCIFDYGVKT    169
                        ***.:**********:*:*:*.*::**:* .:****::*********:**** *** .*:

AtHEXO1                 VQIYKAPWYIQDKPRFGYRGLLIDTSRHYLPIDVIKQIIESMSFAKLNVLHWHIVDEQSF    229
HEXO1A                  VQIHKAPWFIQDKPRFAYRGLLLDTSRHYLPIEIIKQIIESMSYAKLNVLHWHIIDEESF    229
101-03794               VQIHKAPWFIQDKPRFAYRGLLLDTSRHYLPIEIIKQIIESMSYAKLNVLHWHIIDEESF    229
Ctg15557/101-10015      VQIRKAPWFIQDKPRFAYRGLLLDTSRHYLPIEIIKQIIESMSYAKLNVLHWHIIDEESF    229
                        *** ****:*******.*****:*********::*********:**********:**:**

AtHEXO1                 PLETPTYPNLWKGAYSRWERYTVEDASEIVRFAKMRGINVMAEVDVPGHAESWGTGYPDL    289
HEXO1A                  PLEVPSYPNLWKGSYTKWERYTVEDAYEIVDFAKLRGINVMAEVDVPGHAESWGAGYPDL    289
101-03794               PLEVPSYPNLWKGSYTKWERYTVEDAYEIVDFAKLRGINVMAEVDVPGHAESWGAGYPDL    289
Ctg15557/101-10015      PLEVPSYPNLWKGSYTKWERYTVEDAYEIVDFAKMRGINVMAEVDVPGHAESWGAGYPDL    289
                        ***.*:*******:*::********* *** ***:*******************:*****

AtHEXO1                 WPSLSCREPLDVTKNFTFDVISGILADMRKIFPFELFHLGGDEVNTDCWKNTTHVKEWLQ    349
HEXO1A                  WPSPSCKEPLDVSKNYTFDVIAGILADMRKIFPFKLFHLGGDEVNTTCWTTTPHLKQWLQ    349
101-03794               WPSPSCKEPLDVSKNYTFDVIAGILADMRKIFPFKLFHLGGDEVNTTCWTTTPHLKQWLQ    349
Ctg15557/101-10015      WPSPSCKEPLDVSKNYTFDVIAGILADMRKIFPFELFHLGGDECC-----SYPHSVCRLQ    344
                        *** **:*****:**:*****:************:********       .  *    **

AtHEXO1                 GRNFTTKDAYKYFVLRAQQIAISKNWTPVNWEETFSSFGK--DLDPRTVIQNWLVSDICQ    407
HEXO1A                  DHNMTSKDAYEYFVLRAQEIAISHNWTPVNWEETFNTFPS--KLNPQTVVHNWLRGGVCQ    407
101-03794               DHNMTSKDAYEYFVLRAQEIAISHNWTPVNWEETFNTFPS--KLNPQTVVHNWLRGGVCQ    407
Ctg15557/101-10015      DHNMTSKDAYEYFVLRAQEIAISHNWTPVNWLRGGVCQEAVAKGFRCIYSNQGLRGGVCQ    404
                        .:*:*:****:*******:****:******* .         .       :: * ..:**

AtHEXO1                 KAVAKGFRCIFSNQGYWYLDHLDVPWEEVYNTEPLNGIEDPSLQKLVIGGEVCMWGETAD    467
HEXO1A                  AAVAKGFRCIYSNQGFWYLDHLDVPWDKVYYTEPLEGIKSISEQKLLLGGEACMWGETAD    467
101-03794               AAVAKGFRCIYSNQGFWYLDHLDVPWDKVYYTEPLEGIKSISEQKLLLGGEACMWGETAD    467
Ctg15557/101-10015      AAVAKGFRCIYSNQGFWYLDHLDVPWDKVYYTEPLEGIKSISEQKLLLGGEACMWGETAD    464
                         *********:****:**********::** ****:**:. * ***::***.********

AtHEXO1                 TSVVLQTIWPRAAAAAERMWSTREAVSKGNITLTALPRLHYFRCLLNNRGVPAAPVDNFY    527
HEXO1A                  ASDVQQTIWPRAAAVAERLWSDKEATSSTNTTSSALQRLEYFRCLLTRRGVPAAPVTNFY    527
101-03794               ASDVQQTIWPRAAAVAERLWSDKEATSSTNTTSSALQRLEYFRCLLTRRGVPAAPVTNFY    527
Ctg15557/101-10015      ASDVQQTIWPRAAAVAERLWSDKEATSSTNTTSAALQRLEYFRCLLTRRGVPTAPVTNFY    524
                        :* * *********.***:** :**.*. * * :** **.******..****:*** ***

AtHEXO1                 ARRPPLGPGSCYAQ  541
HEXO1A                  ARRPPLGPGSCYEQ  541
101-03794               ARRPPLGPGSCYEQ  541
Ctg15557/101-10015      ARRPPLGPGSCYEQ  538
                        ************ *
```

Figure S1. Amino acid sequence alignment (Clustal Omega) of *A. thaliana* HEXO1, predicted open reading frames (ORFs) of HEXO1 orthologs from the Sol Genomics Network draft genome for *N. benthamiana* (v1.0.1) and retrieved HEXO1 ORFs from Shin and co-workers(Shin et al., 2017). For one predicted ORF the sequences of Niben101Ctg15557g00001.1 (highlighted in orange) and Niben101Scf10015g07014.1 were combined.

```
AtHEXO2            -----------------------MLTLSKF-HVILIPILFFITLLSPLFSIALPINIWP      35
101-09360          --MAY----FISFLSSKQHHKMRGEKTFFSFLPLLLISLL-FFIFISHTTATNYPINIWP      53
NbHEXO2A           --MAY----FISFLSSKQHHKMRGEKTFFSFLPLLLISLL-FFIFISHTTATNYPINIWP      53
101-15928/01151    --MAYFNFKVILFLSSKQHHKMRGEKTFFSFLPLFLVSLL-FFIFISQTTATNYPINVWP      57
NbHEXO2B           MSMAYFNFKVILFLSSKQHHKMRGEKTFFSFLPLFLVSLL-FFILISQTTATNYPINVWP      59
                                            *: .*  ::*: :* *: ::*   :   ***:**

AtHEXO2            KPRFLSWPQHKAIALSPNFTILAPEHQYLSASVTRYHNLIRSENYSPLISYPVKLMKRYT      95
101-09360          KPTTFNWPNPKSIFLSPNFTISHPPHRYLTPAVYRYLHLILSEHHRPIITPAFNLTSSTP      113
NbHEXO2A           KPTTFNWPNPKSIFLSPNFTISHPPHRYLTPAVYRYLHLILSEHHRPIITPAFNLTSSTP      113
101-15928/01151    KPTTFNWPNPKSISLSPNFTISHPPHRYLTPAVYRYLHLILSEHHRPIITPAINLTSYKP      117
NbHEXO2B           KPTTFNWPNPKSISLSPNFTISHPPHRYLTPAVYRYLHLILSEHHRPIITPAINLTSYKP      119
                   **  :.**: *:* *******  * *:**: :* ** :** **:: *:*:  .:* .

AtHEXO2            LRNLVVTVTDFSLPLHHGVDESYKLSIPI-GSFSAHLLAHSAWGAMRGLETFSQMIWGTS      154
101-09360          LRSLIISISDVTSPLAHGVNESYALSTPSDGSPSAYITAETVWGAMRGLETFSQLVYGKP      173
NbHEXO2A           LRSLIISISDVTSPLAHGVNESYALSTPSDGSPSAYITAETVWGAMRGLETFSQLVYGKP      173
101-15928/01151    LHSLIISISDVTSPLAHGINESYALSTPSDGSPSAYITAETVWGAMRGLETFSQLVYGNP      177
NbHEXO2B           LHSLIVSISDVTSPLAHGINESYALSTPSDGSPSAYITAETVWGAMRGLETFSQLVYGNP      179
                   *:.*:::::*.: ** **::*** ** *  ** **:: *.:.*************:::*.

AtHEXO2            PDLCLPVGIYIQDSPLFGHRGVLLDTSRNYYGVDDIMRTIKAMSANKLNVFHWHITDSQS      214
101-09360          TR--VAAGVYIHDLPIFAHRGVMLDTSRNFYGVDDLLRLIKAMSMNKLNVFHWHITDSHS      231
NbHEXO2A           TR--VAAGVYIHDLPIFAHRGVMLDTSRNFYGVDDLLRLIKAMSMNKLNVFHWHITDSHS      231
101-15928/01151    TR--VAAGVYIRDLPIFAHRGVMLDTSRNFYGVDDLLRLIKAMSMNKLNVFHWHITDSHS      235
NbHEXO2B           TR--VAAGVYIRDLPIFAHRGVMLDTSRNFYGVDDLLRLIKAMSMNKLNVFHWHITDSHS      237
                       : .*:**:* *:*.****:******:*****::* ***** *************:*

AtHEXO2            FPLVLPSEPSLAAKGSLGPDMVYTPEDVSKIVQYGFEHGVRVLPEIDTPGHTGSWGEAYP      274
101-09360          FPLVIPSEPELAGKGAYGNEMMYSPADVQKIVEFGLEHGVRVLPEIDMPAHTGSWAEAYP      291
NbHEXO2A           FPLVIPSEPELAGKGAYGNEMMYSPADVQKIVEFGLEHGVRVLPEIDMPAHTGSWAEAYP      291
101-15928/01151    FPLVIPSEPELAGKGAYGNEMMYSPADVQKIVEFGLEHGVRVLPEIDMP-----------      284
NbHEXO2B           FPLVIPSEPELAGKGAYGNEMMYSPADVQKIVGFGLEHGVRVLPEIDMPAHTGSWAEAYP      297
                   ****:****.**.**: * :*:*:* **.*** :*:*********** *

AtHEXO2            EIVTCANMFWWPAGKSWEERLASEPGTGQLNPLSPKTYEVVKNVIQDIVNQFPESFFHGG      334
101-09360          EIITCANMFWWPAASS--PALAAEPGTGQLNPLSPKTYEVAKNVIRDTIAMFPDLLFHGG      349
NbHEXO2A           EIITCANMFWWPAASS--PALAAEPGTGQLNPLSPKTYEVAKNVIRDTIAMFPDLLFHGG      349
101-15928/01151    -------MFWWPAASS--PALAAEPGTGQLNPLSPKTYEVAKNVIHDTIAMFPDLLFHGG      335
NbHEXO2B           EIITCANMFWWPAASS--PALAAEPGTGQLNPLSPKTYEVAKNVIHDTIAMFPDLLFHGG      355
                          ******..*    **:****************.****:* :  **: :****

AtHEXO2            GDEVIPGCWKTDPAINSFLSSGGTLSQLLEKYINSTLPYIVSQNRTVVYWEDVLLDAQIK      394
101-09360          ADEINSACWNTDPSIQKFVTSNGTLSQLLEMFVNNTLPEILSLNRTVVYWEDVILSANVK      409
NbHEXO2A           ADEINSACWNTDPSIQKFVTSNGTLSQLLEMFVNNTLPEILSLNRTVVYWEDVILSANVK      409
101-15928/01151    ADEINSACWNTDPSIQKFVASNGTLSQLLEIFINNTLPEIISLNRTVVYWEDVILSANVK      395
NbHEXO2B           ADEINSACWNTDPSIQKFVASNGTLSQLLEIFINNTLPEIISLNRTVVYWEDVILSANVK      415
                   .**:  .**:***:*:.*::*.******** ::*.*** *:* **********:*.*::*

AtHEXO2            ADPSVLPKEHTILQTWNNGPENTKRIVAAGYRVIVSSSEFYYLDCGHGGFLGNDSIYDQK      454
101-09360          VNPSLLSPQNVIMQTWNNGPNNTKRLVTSGYRVIVSSADYYYLDCGHGSFVGNDSRYEQP      469
NbHEXO2A           VNPSLLSPQNVIMQTWNNGPNNTKRLVTSGYRVIVSSADYYYLDCGHGSFVGNDSRYEQP      469
101-15928/01151    VNPSLLSPENVIMQTWNNGPNNTKRLVTSGYRVVVSSADYYYLDCGHGSFVGNDSRYDQP      455
NbHEXO2B           VNPSLLSPENVIMQTWNNGPNNTKRLVTSGYRVVVSSADYYYLDCGHGSFVGNDSRHDQP      475
                   .:**:*  ::.*:*******:****:*::****:***::********.*:**** ::*

AtHEXO2            ----ESGGGSWCAPFKTWQSIYNYDIADGLLNEEERKLVLGGEVALWSEQADSTVLDSRL      510
101-09360          PGTDQGNGGSWCGPFKTWETIYNYDITYGLTD-EEAKLVIGGEVALCPNKLI-----QLL      523
NbHEXO2A           PGTDQGNGGSWCGPFKTWETIYNYDITYGLTD-EEAKLVIGGEVALCPNKLI-----QLL      523
101-15928/01151    PGTDQGNGGSWCGPFKTWETIYNYDITYGLTD-EEAQLVIGGEVAL--------------      500
NbHEXO2B           PGTDQGNGGSWCGPFKTWETIYNYDITYGLTD-EEAQLVIGGEVALWSEQADSTVMDSRI      534
                       :..*****.*****::******: ** : ** :**:******

AtHEXO2            WPRASALAESLWSGNRDER-GVKRCGEAVDRLNLWRYRMVKRGIGAEPIQPLWCLKNPGM      569
101-09360          WIQESGQEHQQWQKHCGQGIAMKQE-----------------------------------      548

NbHEXO2A           WIQESGQEHQQWQKHCGQGIAMKQE-----------------------------------      548
101-15928/01151    ------------------------------------------------------------      500
NbHEXO2B           WPRGSAMAETLWSGNRDET-GMKRYAEATDRLNEWRYRMVARGIGAEPIQPLWCVKNPGM      593


AtHEXO2            CNTVHGALQDQ       580
101-09360          -----------       548
NbHEXO2A           -----------       548
101-15928/01151    -----------       500
NbHEXO2B           CNTVHPFTS--       602
```

Figure S2. Amino acid sequence alignment (Clustal Omega) of *A. thaliana* HEXO2, predicted open reading frames (ORFs) of HEXO2 orthologs from the Sol Genomics Network draft genome for *N. benthamiana* (v1.0.1) and retrieved HEXO2 ORFs in this study. For one predicted ORF we combined the sequences of Niben101Scf15928g00003.1 and Niben101Scf01151g00003.1.

```
AtHEXO3         MRGSGAKIAGVLPLFMLFIAGTISAFEDIERLRIWPLPAQ----------SGDFKLVTEG      50
101-02405       -----------------MNILVISADANIKNLRIWPMPLSVSYGHRNLQLSNDFVLKTEG      43
NbHEXO3B        MGKLGVQS---ILFAVIVQILVISADANIKNLRIWPMPLSVSYGHRNLQLSNDFVLKTEG      57
101-02017       MGKLGFRS---ILFAAIVQILVVSADANIKNLKIWPMPLSVSYGHRTLLLSNDFVLKTEG      57
NbHEXO3A        MGKLGFRS---ILFAAIVQILVVSADANIKNLKIWPMPLSVSYGHRTLLLSNDFVLKTEG      57
                                 .   .:**  :*:.*:***:* .          *.** * ***

AtHEXO3         SKYGDASGILKEGFDRMLGVVRLSHVISGDRNSSGTGGSALLQGLHVIISSSTDELEYGA      110
101-02405       SKYPDASGILKEGFSRLLDVVKAAHVVDAN--FSYAGPSPVLKGIQVVVLSPSDELQYGV      101
NbHEXO3B        SKYPDASGILKEGFSRLLDVVKAAHVVDAN--FSYAGPSPVLKGIQVVVLSPSDELQYGV      115
101-02017       SKYPDTSGILKEGFSRLLDVVKVAHVVDAN--FSYAGPSPVLKGIQVVVLSPNDELQYGV      115
NbHEXO3A        SKYPDTSGILKEGFSRLLDVVKVAHVVDAN--FSYAGPSPVLKGIQVVVLSPNDELQYGV      115
                *** *:********.*:*.**: :**:..:   * :* * :*:*::*:: * .***:**.

AtHEXO3         DESYKLVVPSPEKPSYAQLEAKSVYGALHGLQTFSQLCHFNLKKKVIEILMTPWNIIDQP      170
101-02405       DESYNLTIPAHGNPAYAHLTAKTVYGALHGLQTFSQVCHFNFTTRTIEVHQVPWSIVDRP      161
NbHEXO3B        DESYNLTIPAHGNPAYAHLTAKTVYGALHGLQTFSQVCHFNFTTRTIEVHQVPWSIVDRP      175
101-02017       DESYNLTIPAHGNPAYAYLTAKTVHGALHGLQTFSQVCHFNFTTRTIEVHQVPWTIVDRP      175
NbHEXO3A        DESYNLTIPAHGNPAYAYLTAKTVHGALHGLQTFSQVCHFNFTTRTIEVHQVPWTIVDRP      175
                ****:*.:*:  :*:** * **:*:***********:****:..:.**:  .**.*:*:*

AtHEXO3         RFSYRGLLIDTSRHYLPLPVIKNVIDSMTYAKLNVLHWHIVDTQSFPLEIPSYPKLWNGA      230
101-02405       RFSYRGLLIDTSRHYLPFPVILKVIDSMAYAKLNVLHWHIVDTQSFPLEIPSFPRLWNGA      221
NbHEXO3B        RFSYRGLLIDTSRHYLPFPVILKVIDSMAYAKLNVLHWHIVDTQSFPLEIPSFPRLWNGA      235
101-02017       RFSYRGLLIDTSRHYLPLPVILKVIDSMAYAKLNVLHWHIVDTQSFPLEIPSFPKLWNGA      235
NbHEXO3A        RFSYRGLLIDTSRHYLPLPVILKVIDSMAYAKLNVLHWHIVDTQSFPLEIPSFPKLWNGA      235
                *****************:*** :*****:***********************:*:*****

AtHEXO3         YSSSQRYTFEDAAEIV----NYARRRGIHVLAEIDVPGHALSWGKGYPALWPSKNCQEPL      286
101-02405       YSSSERYTVVDAAEIVRQSCRYAGRRGINVLAELDVPGHAQSWGTGYPSLWPSKDCQQPL      281
NbHEXO3B        YSSSERYTVVDAAEIV----RYAGRRGINVLAELDVPGHAQSWGTGYPSLWPSKDCQQPL      291
101-02017       YSSSERYTVTDAAEIVRQSCRYAGRRGINVLAELDVPGHAQSWGNGYPSLWPSKDCQQPL      295
NbHEXO3A        YSSSERYTVTDAAEIV----RYAGRRGINVLAELDVPGHAQSWGNGYPSLWPSKDCQQPL      291
                ****:***. ******    .** ****:****:****** ***.***:*****:**:**

AtHEXO3         DVSSDFTFKVIDGILSDFSKIFKFKFVHLGGDEVNTTCWSAT------------------      328
101-02405       DISNDFTFKLIDGILSDFSKIFKYRFVHLGGDEVDTIVIPGDKNTGRCVCHYMNSSKLQF      341
NbHEXO3B        DISNDFTFKLIDGILSDFSKIFKYRFVHLGGDEVDTSCWTLT------------------      333
101-02017       DISNDFTFKVIDGILSDFSKIFKYRFVHLGGDEVDTSKLTCSDNCPDLVVSIFLCDTLLF      355
NbHEXO3A        DISNDFTFKVIDGILSDFSKIFKYRFVHLGGDEVDTSCWTL-------------------      332
                *:*.*****:*************::*********:*

AtHEXO3         ----PRIAQWLKKHRMSEKEAYQYFVLRAQKIALSHGYEIINWEETFINFGSKLNRKTVV      384
101-02405       RSSHFHLGGMLKQNKLNGTTAYEYFVLRAQKIALSHGYEIINWEETFNNFGNKLIPNTIV      401
NbHEXO3B        ----PRIRKWLKQNKLNGTTAYEYFVLRAQKIALSHGYEIINWEETFNNFGNKLIPNTIV      389
101-02017       PW-TKTLVDGLRQNKLNRTTAYEYFVLRAQKIALSHGYEIINWEETFNNFGNKLSRNSIV      414
NbHEXO3A        ---TPRIRKWLRQNKLNRTTAYEYFVLRAQKIALSHGYEIINWEETFNNFGNKLSRNSIV      389
                      :   *:::::. . **:************************ ***.**  :::*

AtHEXO3         HNW----------------------------------------LNTGLVENVTASGLRCI      404
101-02405       HNWCVLL------------------------------------LGGGVAQQVTAAGLRCI      425
NbHEXO3B        HNW----------------------------------------LGGGVAQQVTAAGLRCI      409
101-02017       HNWCVLLSCCISWKLHISSYLFSRLFHFLYSSLPIKVIEKVRKLGGGVAQQVTAAGLRCI      474
NbHEXO3A        HNW----------------------------------------LGGGVAQQVTAAGLRCI      409
                ***                                        *. *:.::***:*****

AtHEXO3         VSNQEFWYLDHIDAPWQGFYANEPFQNITDKKQQSLVLGGEVCMWGEHIDASDIEQTIWP      464
101-02405       VSNQDKWYLDHIDTTWQDFYSNEPLTNIRNPKQQRLVIGGEVCMWGEHIDGSNIETTIWP      485
NbHEXO3B        VSNQDKWYLDHIDTTWQDFYSNEPLTNIRNPKQQRLVIGGEVCMWGEHIDGSNIETTIWP      469
101-02017       VSNQDKWYLDHIDTTWQDFYSNEPLTNITNPKQQRLVIGGEVCMWGEHIDGSNIETTIWP      534
NbHEXO3A        VSNQDKWYLDHIDTTWQDFYSNEPLTNITNPKQQRLVIGGEVCMWGEHIDGSNIETTIWP      469
                ****: *******: **.**:***: ** : *** **:************.*:** ****

AtHEXO3         RAAAAAERLWTPYAKLAKNPNNVTTRLAHFRCLLNQRGVAAAPLVGGGRVVPFEPGSCLA      524
101-02405       RAAAAAERLWTAYDNLAKNPSQVTRRLAHFRCLLNQRGVASVPLTGGGRAAPEDPADVTR      545
NbHEXO3B        RAAAAAERLWTAYDNLAKNPSQVTRRLAHFRCLLNQRGVASVPLTGGGRAAPEDPGSCYQ      529
101-02017       RAAAAAERLWTAYDNLAKNPSQVTRRLAHFRCLLNRRGVASGPLTGGGRAAPEDPGSCYQ      594
NbHEXO3A        RAAAAAERLWTAYDNLAKNPSQVTRRLAHFRCLLNRRGVASGPLTGGGRAAPEDPGSCYQ      529
                *********** * :*****.:** **********:****: **.****..* :*..
```

Figure S3. Amino acid sequence alignment (Clustal Omega) of *A. thaliana* HEXO3, predicted open reading frames (ORFs) of HEXO3 orthologs from the Sol Genomics Network draft genome for *N. benthamiana* (v1.0.1) and retrieved HEXO3 ORFs from Shin and co-workers(Shin et al., 2017) and this study.

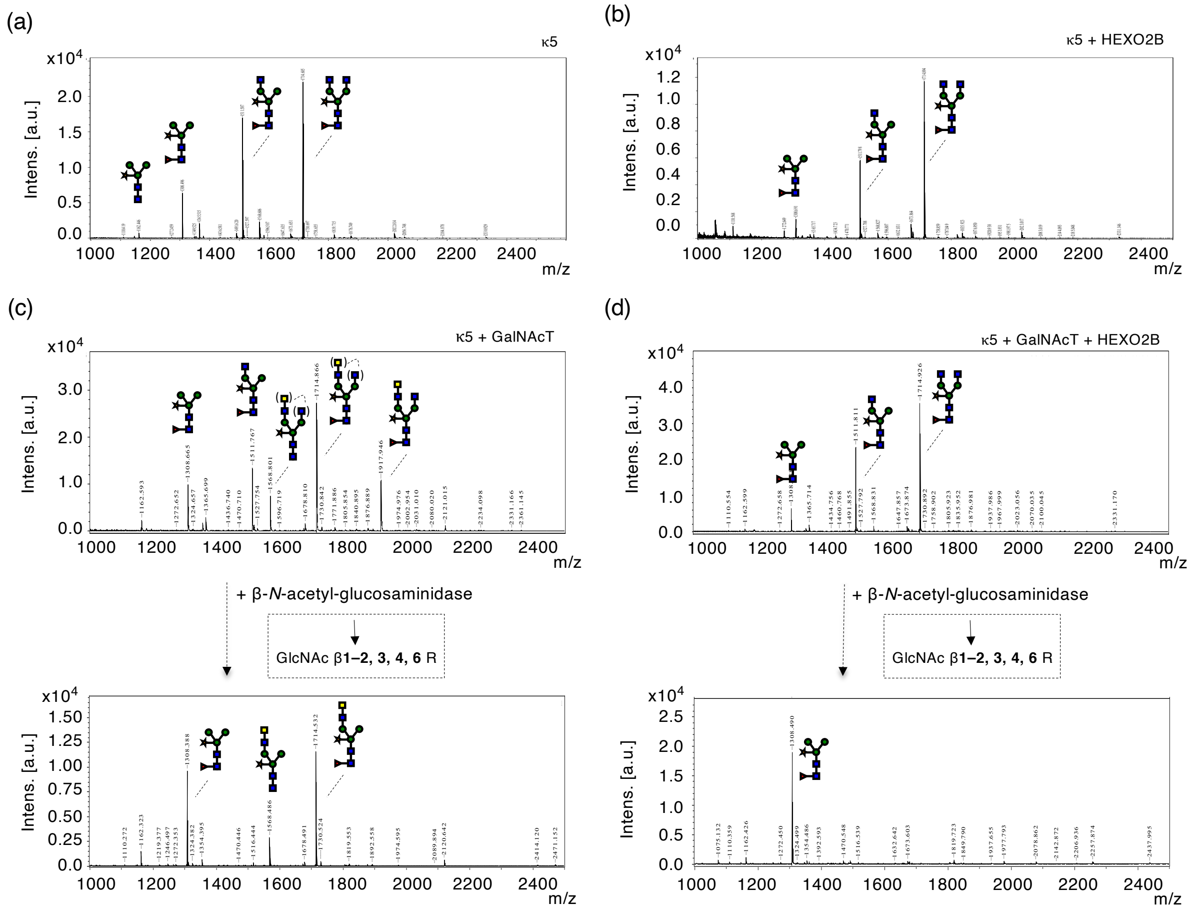

Figure S4. HEXO2B substrate specificity. Kappa-5 was co-expressed with p19 and combinations of HEXO2B and/or CeGalNAcT in wild-type plants. Kappa-5 was purified from the apoplast fluid (at 6 dpi) and the N-glycan composition of kappa-5 was analyzed MALDI-TOF-MS. MS profiles are given for N-glycans of wild-type kappa-5 (a), kappa-5 with HEXO2B (b), kappa-5 with CeGalNAcT (c) and kappa-5 with CeGalNAcT and HEXO2B (d). To distinguish terminal GlcNAc and GalNAc residues we treated the N-glycans with glucosaminidase for which the substrate specificity is indicated in the dashed box. When a MS peak represents multiple N-glycan structures of identical mass, the possible positions of these sugar residues on the N-glycan are indicated between brackets.

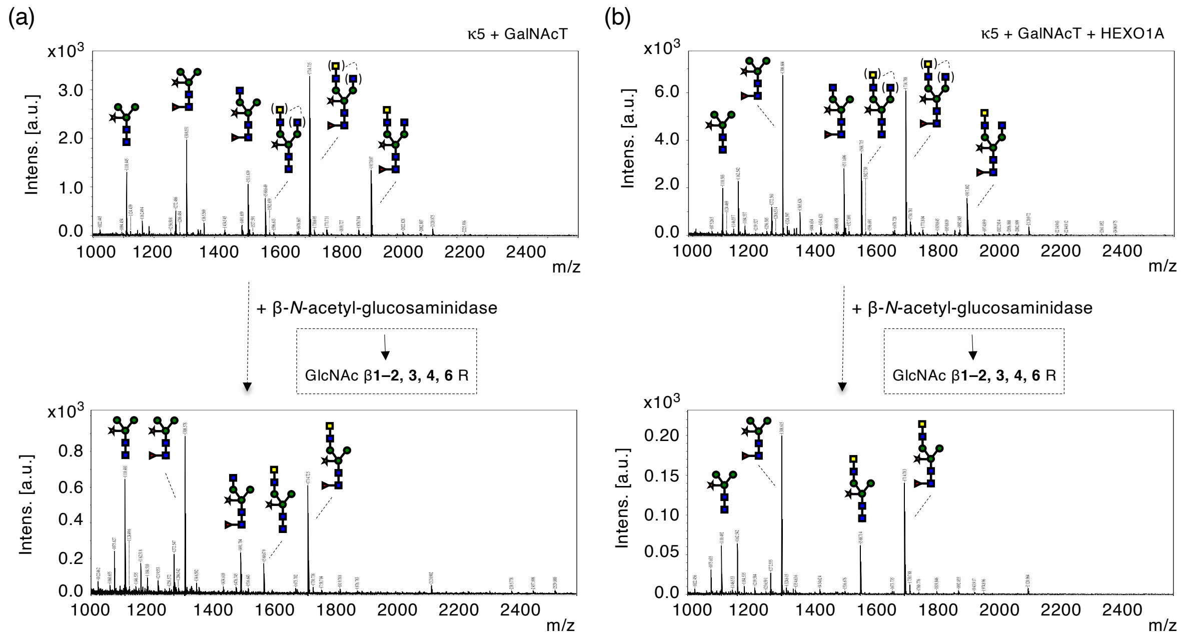

Figure S5. HEXO1A substrate specificity. Kappa-5 was co-expressed with p19, CeGalNAcT and with/without HEXO1A in wild-type plants. Kappa-5 was purified from the apoplast fluid (at 6 dpi) and the N-glycan composition of kappa-5 was analyzed MALDI-TOF-MS. MS profiles are given for N-glycans of kappa-5 with CeGalNAcT (a) and kappa-5 with CeGalNAcT and HEXO1A (b). To distinguish terminal GlcNAc and GalNAc residues we treated the N-glycans with glucosaminidase for which the substrate specificity is indicated in the dashed box. When a MS peak represents multiple N-glycan structures of identical mass, the possible positions of these sugar residues on the N-glycan are indicated between brackets.

Figure S6. Nucleotide sequence alignment (Clustal Omega) of HEXO2A, HEXO2B and the target region of the Δ2 hairpin construct.

```
CLUSTAL O(1.2.4) multiple sequence alignment


HEXO2A      ------ATGGCTTACTT------------CATTTCATTTCTCTCCTCAAAACAACACCAC      42
HEXO2B      ATGAGCATGGCTTACTTCAACTTCAAAGTCATTTTATTTCTCTCCTCAAAACAACACCAC      60
hairpin     ------------------------------------------------------------      0


HEXO2A      AAAATGAGAGGAGAGAAAACATTTTTCTCCTTTCTTCCACTACTCCTTATCTCCTTGTTA      102
HEXO2B      AAAATGAGAGGAGAGAAAACATTTTTCTCCTTTCTTCCACTATTCCTTGTCTCCTTGTTA      120
hairpin     ------------------------------------------------------------      0


HEXO2A      TTCTTCATCTTCATTTCTCACACAACAGCTACAAATTACCCAATCAATATCTGGCCCAAG      162
HEXO2B      TTCTTTATCTTCATTTCTCAGACAACAGCTACAAATTACCCAATCAATGTCTGGCCCAAG      180
hairpin     ------------------------------------------------------------      0


HEXO2A      CCCACAACATTCAATTGGCCCAACCCAAAATCCATCTTCCTCTCCCCAAACTTCACCATC      222
HEXO2B      CCCACAACATTCAATTGGCCCAACCCAAAATCCATCTCCCTCTCTCCAAACTTCACCATC      240
hairpin     -------------------------------CCATCTCCCTCTCTCCAAACTTCACCATC      29
                                           ****** ****** ***************

HEXO2A      TCCCACCCACCCCACCGTTATCTCACTCCCGCGGTTTACCGTTATCTCCACCTCATCCTC      282
HEXO2B      TCCCACCCACCCCACCGTTACCTCACTCCCGCCGTTTACCGTTATCTCCACCTCATCCTC      300
hairpin     TCCCACCCACCCCACCGTTACCTCACTCCCGCCGTTTACCGTTATCTCCACCTCATCCTC      89
            ******************** *********** ***************************

HEXO2A      TCCGAGCACCACCGTCCGATCATCACTCCGGCTTTTAACCTGACGTCATCCACGCCGTTA      342
HEXO2B      TCCGAGCACCACCGACCGATCATCACTCCGGCGATTAACCTGACGTCATACAAGCCGTTA      360
hairpin     TCCGAGCACCACCGACCGATCATCACTCCGGCGATTAACCTGACGTCATACAAGCCGTTA      149
            ************** *****************  *************** ** *******

HEXO2A      CGTAGCCTCATCATCTCTATCTCCGACGTCACTTCACCCCTTGCTCACGGAGTCAACGAA      402
HEXO2B      CATAGCCTCATCATCTCCATCTCCGATGTCACTTCACCCCTTGCTCACGGAATCAACGAA      420
hairpin     CATAGCCTCATCATCTCCATCTCCGATGTCACTTCACCCCTTGCTCACGGAATCAACGAA      209
            * *************** ******** ************************ ********

HEXO2A      TCCTATGCTCTCTCCACTCCTTCCGACGGCTCTCCCTCCGCCTATATCACCGCGGAAACT      462
HEXO2B      TCCTATGCTCTCTCCACTCCTTCCGACGGaTCcCCgTCCGCCTATATCACCGCCGAAACA      480
hairpin     TCCTATGCTCT-------------------------------------------------      220
            ***********

HEXO2A      GTATGGGGAGCAATGCGAGGTCTCGAGACTTTCTCGCAACTCGTGTACGGAAAACCTACC      522
HEXO2B      GTATGGGGGGCCATGCGAGGTCTCGAGACTTTCTCGCAACTCGTGTACGGAAACCCTACC      540
hairpin     ------------------------------------------------------------      220


HEXO2A      AGAGTCGCCGCCGGCGTGTACATACACGATCTGCCGATTTTCGCGCACCGAGGTGTGATG      582
HEXO2B      AGAGTCGCCGCCGGCGTGTACATACGCGATCTGCCGATTTTCGCGCACCGAGGTGTGATG      600
hairpin     ------------------------------------------------------------      220


HEXO2A      CTGGACACTTCGAGAAACTTTTACGGCGTTGATGATTTGTTGAGGCTTATTAAAGCTATG      642
HEXO2B      CTGGACACTTCGAGAAACTTCTACGGCGTCGATGATTTATTGAGGCTTATTAAAGCTATG      660
hairpin     ------------------------------------------------------------      220


HEXO2A      AGTATGAACAAGTTGAATGTTTTCCACTGGCACATAACTGATTCACATTCGTTTCCGCTT      702
HEXO2B      AGTATGGACAAGTTGAATGTTTTCCACTGGCACATAACTGATTCACATTCGTTTCCGCTT      720
hairpin     ------------------------------------------------------------      220


HEXO2A      GTGATTCCTTCGGAGCCGGAGCTCGCCGGAAAAGGAGCATACGGCAATGAGATGATGTAC      762
HEXO2B      GTGATTCCTTCGGAGCCGGAGCTCGCCGGAAAAGGAGCATACGGCAATGAGATGATGTAC      780
hairpin     ------------------------------------------------------------      220


HEXO2A      TCGCCGGCGGACGTGCAGAAGATCGTGGAATTTGGACTGGAACACGGAGTTAGAGTTTTA      822
HEXO2B      TCGCCGGCAGACGTGCAGAAGATCGTGGAATTTGGACTGGAACATGGAGTTAGAGTTTTA      840
hairpin     ------------------------------------------------------------      220


HEXO2A      CCTGAAATTGACATGCCTGCACATACAGGATCATGGGCTGAAGCTTACCCTGAGATTATC      882
HEXO2B      CCTGAAATTGACATGCCTGCACATACAGGATCATGGGCTGAAGCTTACCCTGAGATTATC      900
hairpin     ------------------------------------------------------------      220


HEXO2A      ACTTGTGCAAATATGTTCTGGTGGCCTGCTGCAAGTAGTCCAGCTCTTGCAGCTGAACCA      942
HEXO2B      ACTTGTGCAAATATGTTCTGGTGGCCTGCTGCAAGTAGTCCAGCTCTTGCAGCTGAACCA      960
hairpin     ------------------------------------------------------------      220


HEXO2A      GGTACTGGTCAACTGAACCCCTTGAGTCCCAAGACCTATGAAGTAGCCAAGAATGTCATC      1002
HEXO2B      GGTACTGGCCAGCTGAACCCCCTGAGTCCCAAGACCTATGAAGTAGCCAAGAATGTCATC      1020
hairpin     ------------------------------------------------------------      220


HEXO2A      CGCGATACTATCGCCATGTTTCCAGATTTGCTCTTTCATGGGGGAGCAGATGAGATCAAT      1062
HEXO2B      CACGATACTATCGCCATGTTTCCAGATTTGCTCTTTCACGGGGGAGCAGATGAGATCAAT      1080
hairpin     ------------------------------------------------------------      220


HEXO2A      TCAGCCTGTTGGAATACTGATCCATCAATCCAAAAGTTTGTCACTAGCAATGGAACTCTC      1122
HEXO2B      TCAGCATGTTGGAATACTGATCCATCAATCCAAAAGTTTGTCGCTAGCAATGGAACTCTC      1140
hairpin     ------------------------------------------------------------      220


HEXO2A      AGTCAGCTACTCGAAATGTTTGTCAATAATACTTTACCTGAAATCCTCTCACTCAACCGT      1182
HEXO2B      AGTCAGCTACTCGAAATCTTTATCAATAATACCTTACCTGAAATCATCTCACTCAACCGT      1200
hairpin     ------------------------------------------------------------      220


HEXO2A      ACGGTGGTCTACTGGGAGGATGTTATATTGAGTGCTAATGTGAAAGTGAATCCATCTCTG      1242
HEXO2B      ACGGTGGTCTATTGGGAGGATGTTATATTGAGTGCTAATGTGAAAGTGAATCCGTCTCTG      1260
hairpin     ------------------------------------------------------------      220


HEXO2A      CTTTCTCCACAGAATGTTATTATGCAAACTTGGAATAATGGACCAAACAATACTAAGCGG      1302
HEXO2B      CTTTCTCCAGAGAATGTTATTATGCAAACTTGGAATAACGGGCCAAATAATACTAAGCGG      1320
hairpin     ------------------------------------------------------------      220


HEXO2A      CTTGTCACTTCTGGCTACCGTGTCATTGTGTCATCTGCAGATTACTATTACTTGGATTGT      1362
HEXO2B      CTTGTCACTTCTGGCTACCGTGTCGTTGTGTCATCTGCAGATTACTATTACTTGGATTGT      1380
hairpin     ------------------------------------------------------------      220


HEXO2A      GGTCATGGGAGCTTCGTTGGAAATGACAGCCGCTATGAGCAACCACCAGGTACTGACCAA      1422
HEXO2B      GGCCATGGTAGCTTCGTTGGAAATGACAGCCGCTATGACCAGCCGCCAGGTACTGACCAA      1440
hairpin     ------------------------------------------------------------      220


HEXO2A      GGCAATGGTGGATCCTGGTGCGGACCTTTCAAGACCTGGGAAACCATATACAACTATGAT      1482
HEXO2B      GGCAATGGTGGATCCTGGTGTGGACCTTTCAAGACCTGGGAAACCATATATAACTACGAT      1500
hairpin     ------------------------------------------------------------      220


HEXO2A      ATAACCTATGGCCTGACTGATGAGGAGGCTAAATTGGTAATTGGAGGGGAAGTAGCATTA      1542
HEXO2B      ATAACCTATGGCCTGACTGATGAGGAGGCTCAATTGGTAATTGGAGGGGAAGTAGCATTA      1560
hairpin     ------------------------------------------------------------      220


HEXO2A      TG-TCCGAACAAGCTGATTCAACTGTTATGGATTCAAGAATCTGGCCAAGAGCATCAGCA      1601
HEXO2B      TGGTCTGAACAAGCTGATTCAACTGTTATGGATTCAAGAATCTGGCCAAGAGGATCAGCA      1620
hairpin     ------------------------------------------------------------      220


HEXO2A      ATGGCAGAAACATTGTGGTCAGGGAATCGCGATGAAACAGGAATGA--------------      1647
HEXO2B      ATGGCAGAAACATTGTGGTCAGGGAATCGTGATGAAACAGGAATGAAGAGATATGCAGAG      1680
hairpin     ------------------------------------------------------------      220


HEXO2A      ------------------------------------------------------------      1647
HEXO2B      GCTACTGATCGACTGAATGAATGGAGGTACAGAATGGTTGCCAGGGGAATAGGTGCTGAA      1740
hairpin     ------------------------------------------------------------      220


HEXO2A      ------------------------------------------------------------      1647
HEXO2B      CCGATTCAGCCTCTGTGGTGTGTCAAAAACCCAGGCATGTGTAACACAGTTCATCCATTT      1800
hairpin     ------------------------------------------------------------      220


HEXO2A      ---------  1647
HEXO2B      ACCAGCTGA  1809
hairpin     ---------  220
```

Figure S7. Nucleotide sequence alignment (Clustal Omega) of HEXO3A, HEXO3B and the target region of the Δ3 hairpin construct.

```
CLUSTAL O(1.2.4) multiple sequence alignment


HEXO3B        ATGGGGAAGTTAGGAGTGCAGAGCATATTATTTGCAGTTATCGTTCAGATTCTAGTAATA      60
HEXO3A        ATGGGGAAGTTAGGATTCCGGAGCATATTATTTGCAGCTATCGTTCAGATTCTAGTAGTA      60
hairpin       ------------------------------------------------------------      0


HEXO3B        TCAGCAGATGCTAATATTAAGAATTTGAGAATATGGCCGATGCCACTATCGGTTAGCTAT      120
HEXO3A        TCAGCAGATGCTAATATTAAGAATTTGAAGATATGGCCGATGCCACTATCAGTTAGCTAT      120
hairpin       ------------------------------------------------------------      0


HEXO3B        GGACATAGGAATCTTCAACTTAGTAATGATTTTGTGCTCAAGACTGAAGGCAGCAAGTAC      180
HEXO3A        GGACATAGGACTCTTCTACTTAGTAATGATTTTGTGCTCAAGACTGAAGGCAGCAAGTAC      180
hairpin       ------------------------------------------------------------      0


HEXO3B        CCTGATGCCTCTGGAATTCTCAAGGAAGGTTTTTCAAGGCTACTTGATGTCGTTAAGGCA      240
HEXO3A        CCTGATACCTCTGGAATTCTTAAAGAAGGTTTTTCTAGGTTACTTGATGTCGTTAAGGTA      240
hairpin       ------------------------------------------------------------      0


HEXO3B        GCTCATGTTGTTGATGCCAACTTCTCTTATGCCGGCCCCTCTCCAGTGCTTAAGGGAATT      300
HEXO3A        GCTCATGTCGTTGACGCCAACTTCTCATATGCCGGCCCCTCTCCAGTGCTAAAGGGAATT      300
hairpin       ------------------------------------------------------------      0


HEXO3B        CAAGTGGTTGTCCTTTCTCCAAGTGATGAGCTACAATATGGTGTTGATGAGTCCTACAAC      360
HEXO3A        CAAGTGGTTGTCCTTTCTCCAAATGATGAGCTACAATATGGTGTTGATGAGTCCTACAAC      360
hairpin       ------------------------------------------------------------      0


HEXO3B        TTGACCATACCAGCACATGGAAACCCAGCCTATGCACATTTAACGGCAAAAACAGTTTAT      420
HEXO3A        TTGACCATACCAGCACATGGAAACCCAGCCTATGCATATTTGACGGCAAAAACAGTTCAT      420
hairpin       ---------------------------------------------GCAAAAACAGTTCAT      15
                                                           ************ **

HEXO3B        GGGGCTTTGCATGGTTTGCAGACATTTAGTCAAGTATGCCATTTTAACTTTACAACCAGA      480
HEXO3A        GGGGCGTTGCATGGTTTGCAGACATTTAGTCAAGTATGCCATTTTAACTTTACAACCAGA      480
hairpin       GGGGCGTTGCATGGTTTGCAGACATTTAGTCAAGTATGCCATTTTAACTTTACAACCAGA      75
              ***** ******************************************************

HEXO3B        ACAATTGAAGTTCATCAAGTTCCGTGGAGCATAGTTGATCGACCAAGATTCTCTTATCGA      540
HEXO3A        ACAATTGAAGTTCATCAAGTTCCATGGACCATAGTTGATCGACCAAGATTCTCTTATCGA      540
hairpin       ACAATTGAAGTTCATCAAGTTCCATGGACCATAGTTGATCGACCAAGATTCTCTTATCGA      135
              *********************** **** *******************************

HEXO3B        GGGCTTTTAATTGATACTTCCCGTCACTATCTGCCATTTCCTGTGATATTGAAGGTTATT      600
HEXO3A        GGGCTTTTAATTGATACTTCCCGTCACTATCTGCCGTTGCCTGTGATATTGAAGGTTATC      600
hairpin       GGGCTTTTAATTGATACTTCCCGTCACTATCTGCCGTTGCCTGTGATATTGAAGGTTATC      195
              *********************************** ** ********************

HEXO3B        GATTCGATGGCTTATGCAAAACTGAATGTGCTGCACTGGCACATTGTAGATACACAATCA      660
HEXO3A        GATTCAATGGCTTATGCAAAACTGAATGTGCTGCACTGGCACATTGTAGATACACAATCA      660
hairpin       GATTCAATGGCTTATGCAAAACTGA-----------------------------------      220
              ***** *******************

HEXO3B        TTCCCACTTGAGATACCTTCATTTCCGAGACTGTGGAATGGTGCTTACTCAAGTTCAGAG      720
HEXO3A        TTCCCACTTGAGATACCTTCATTTCCAAAACTGTGGAATGGTGCTTACTCAAGTTCAGAG      720
hairpin       ------------------------------------------------------------      220


HEXO3B        CGGTACACTGTAGTTGATGCTGCTGAGATTGTAAGATATGCAGGAAGACGTGGAATCAAT      780
HEXO3A        CGGTACACTGTAACTGATGCTGCTGAGATTGTAAGATATGCAGGAAGACGTGGAATCAAT      780
hairpin       ------------------------------------------------------------      220


HEXO3B        GTATTAGCTGAATTAGACGTTCCAGGACATGCTCAATCTTGGGGTACTGGTTATCCTTCT      840
HEXO3A        GTATTAGCTGAATTAGACGTTCCGGGACATGCTCAATCTTGGGGTAATGGTTATCCTTCT      840
hairpin       ------------------------------------------------------------      220


HEXO3B        TTATGGCCGTCAAAAGATTGTCAACAGCCACTTGACATAAGCAATGATTTCACTTTCAAA      900
HEXO3A        TTATGGCCGTCAAAAGATTGTCAACAGCCACTTGACATAAGCAATGATTTCACTTTCAAA      900
hairpin       ------------------------------------------------------------      220


HEXO3B        CTCATAGACGGAATTCTCTCAGATTTTAGCAAGATATTCAAATACAGATTTGTTCATCTA      960
HEXO3A        GTAATAGACGGAATTCTTTCAGATTTTAGCAAGATATTCAAGTACAGATTTGTTCATCTA      960
hairpin       ------------------------------------------------------------      220


HEXO3B        GGAGGTGATGAAGTGGATACAAGTTGCTGGACGTTAACTCCTCGTATAAGAAAGTGGTTG      1020
HEXO3A        GGAGGTGATGAAGTGGATACAAGTTGCTGGACGTTAACTCCTCGTATAAGAAAGTGGTTG      1020
hairpin       ------------------------------------------------------------      220


HEXO3B        AAGCAAAACAAGTTGAATGGAACCACTGCTTATGAGTACTTCGTCTTGAGAGCACAGAAG      1080
HEXO3A        AGGCAAAACAAGTTGAATAGAACCACTGCTTATGAGTACTTCGTCTTGCGAGCACAGAAG      1080
hairpin       ------------------------------------------------------------      220


HEXO3B        ATAGCTTTATCTCATGGATATGAAATTATAAACTGGGAAGAGACGTTCAATAACTTTGGA      1140
HEXO3A        ATAGCTTTATCTCATGGATATGAAATTATAAACTGGGAAGAGACGTTCAATAACTTTGGA      1140
hairpin       ------------------------------------------------------------      220


HEXO3B        AATAAGTTGATCCCAAACACTATAGTTCACAACTGGCTCGGGGGCGGTGTTGCTCAGCAA      1200
HEXO3A        AATAAGTTGAGCCGAAATTCTATAGTTCACAACTGGCTCGGGGGCGGTGTTGCTCAGCAA      1200
hairpin       ------------------------------------------------------------      220


HEXO3B        GTAACTGCAGCTGGATTGCGGTGCATTGTTAGCAACCAGGACAAGTGGTATTTGGACCAT      1260
HEXO3A        GTAACTGCAGCTGGATTGCGGTGCATTGTTAGCAACCAGGACAAGTGGTATTTGGACCAT      1260
hairpin       ------------------------------------------------------------      220


HEXO3B        ATAGATACCACATGGCAGGATTTCTATTCAAATGAGCCACTAACTAATATTAGAAATCCC      1320
HEXO3A        ATAGACACTACATGGCAGGATTTCTATTCAAATGAGCCACTAACTAATATTACAAATCCC      1320
hairpin       ------------------------------------------------------------      220


HEXO3B        AAGCAACAACGGCTGGTTATTGGGGGTGAGGTATGTATGTGGGGTGAGCATATTGATGGA      1380
HEXO3A        AAGCAACAACGGCTGGTTATTGGGGGTGAGGTATGTATGTGGGGTGAGCATATTGATGGA      1380
hairpin       ------------------------------------------------------------      220


HEXO3B        TCAAACATCGAAACAACCATATGGCCCCGTGCAGCAGCAGCTGCAGAGAGGCTATGGACT      1440
HEXO3A        TCAAACATTGAAACGACCATATGGCCGCGTGCAGCAGCAGCTGCAGAGAGGCTGTGGACT      1440
hairpin       ------------------------------------------------------------      220


HEXO3B        GCTTATGACAACCTTGCCAAGAATCCAAGTCAAGTTACTCGCAGGCTGGCTCATTTTAGG      1500
HEXO3A        GCTTATGACAACCTTGCCAAGAATCCAAGTCAAGTTACTCGCAGGTTGGCTCATTTTAGG      1500
hairpin       ------------------------------------------------------------      220


HEXO3B        TGCCTATTGAATCAAAGAGGAGTGGCTTCTGTTCCATTAACTGGAGGCGGGCGAGCTGCA      1560
HEXO3A        TGCCTATTGAATCGAAGAGGAGTGGCTTCTGGTCCTTTAACCGGAGGCGGGCGAGCTGCA      1560
hairpin       ------------------------------------------------------------      220


HEXO3B        CCAGAAGATCCAGGTTCTTGCTATCAGCAATAG  1593
HEXO3A        CCAGAAGATCCAGGTTCTTGCTATCAGCAATAG  1593
hairpin       ---------------------------------  220
```
